# Immune Response Gene Polymorphisms and Cervical Cancer Susceptibility

**DOI:** 10.3390/ijms27209384

**Published:** 2026-10-21

**Authors:** Roksana Dwornik, Dagmara Cylwik, Katarzyna Białkowska

**Affiliations:** Department of Genetics and Pathology, International Hereditary Cancer Center, Pomeranian Medical University, 71-252 Szczecin, Poland

**Keywords:** Cervical cancer, Human papillomavirus (HPV), Immune response gene polymorphisms

## Abstract

Cervical cancer (CxCa) remains one of the most common malignancies in women worldwide. Although almost all cases are associated with chronic infection with oncogenic human papillomavirus (HPV) types, not every infected woman develops the disease, indicating a significant role of genetic factors. This study reviewed 131 studies on genes related to immune system function and their impact on the risk of developing cervical cancer. The majority of the studies analyzed were conducted in Asian populations, primarily Chinese and Indian. The analysis focused in particular on polymorphisms in genes involved in the antiviral response, inflammatory processes, antigen presentation, and regulation of lymphocyte activity. Most of the evidence points to the importance of human leukocyte antigens (HLA) genes, Toll-like receptors (TLRs), interleukins (ILs), tumor necrosis factor alpha (TNF-α), and interferons (IFNs). Variants in these genes may influence the effectiveness of HPV clearance, susceptibility to persistent infection, and the risk of progression of precancerous lesions to invasive cancer. It is worth emphasizing that identifying genetic risk factors may, in the future, support the development of personalized prevention strategies, screening tests, and the assessment of the individual risk of disease. Nevertheless, most available findings warrant further validation in large and diverse populations. In summary, cervical cancer is the result of a complex interaction between HPV infection and the host’s genetic predisposition. Research on immune response genes provides valuable information about the mechanisms of disease development, and may contribute to improved methods of prevention and early detection.

## 1. Introduction

Cervical cancer (CxCa) is one of the leading causes of mortality among women worldwide, although its incidence varies significantly by region. In developed countries, the risk of both incidence and death is lower than in developing countries [1]. In 2022, an estimated 662,044 new cases and 384,709 deaths were reported globally [2]. The highest rates are observed in Sub-Saharan Africa, Latin America, and South and Southeast Asia, while the lowest occur in Western Europe, Australia, and North America [3].

The main risk factors for cervical cancer include infection with oncogenic types of human papillomavirus (HPV), co-infection with other sexually transmitted diseases, human immunodeficiency virus (HIV) infection, early age at sexual initiation, high number of pregnancies, use of hormonal contraception, and tobacco smoking [4,5,6].

HPV is responsible for nearly all cases of cervical cancer. Prevention primarily includes vaccination and HPV screening programs [5]. There are many types of this virus; however, HPV types 16 and 18 account for the majority of cases [1]. Among the numerous variants, approximately 15 are classified as high-risk oncogenic types, while some of the remaining types are considered potentially oncogenic [7,8,9].

Most HPV infections resolve spontaneously within 1–2 years. However, persistent infections with high-risk types may lead to the development of precancerous lesions known as cervical intraepithelial neoplasia (CIN) [6]. CIN1 lesions usually regress spontaneously and rarely progress to cancer. In contrast, CIN2 and especially CIN3 have a higher potential for progression, with CIN3 considered a precancerous condition [10]. The development of cervical cancer is a long-term process and typically takes around 20 years [6].

Genome-wide association studies (GWAS) indicate a significant role of genetic factors in the development of cervical cancer. Among others, single-nucleotide polymorphisms (SNPs) have been analyzed for their association with seropositivity to HPV16 and other viral types. These findings suggest that genetic factors may influence both susceptibility to HPV infection and the risk of disease progression [1]. Most available studies have been conducted in Asian populations, which may be due to their large population size and relatively high disease prevalence. In 2022, GLOBOCAN estimates indicate that China and India together accounted for approximately 42% of global CxCa cases and 39% of cervical cancer-related deaths, highlighting the substantial contribution of these populations to the global disease burden [2].

Among the identified genetic variants, genes related to immune system function are of particular interest. Variations in these genes have been shown to influence not only the risk of developing cervical cancer but also the progression of the disease and survival outcomes. The local immune response is therefore considered a key factor in cervical carcinogenesis following persistent infection with high-risk oncogenic HPV [11].

The aim of this narrative review is to synthesize and summarize the current knowledge on cervical cancer, with particular emphasis on the role of genetic factors in its development. It is also important to emphasise that, due to the large number of studies available, this review focuses specifically on research concerning genes related to immunological processes.

## 2. Methodology

The literature search for this narrative review was conducted in January 2026, and articles published between 1993 and January 2026 were included in the review. The search strategy was based on the following keywords: ‘genetic susceptibility’, ‘cervical cancer’ and ‘immunity’. Boolean operators were used to combine the search terms. The literature search was performed using the PubMed database.

**Figure 1:**
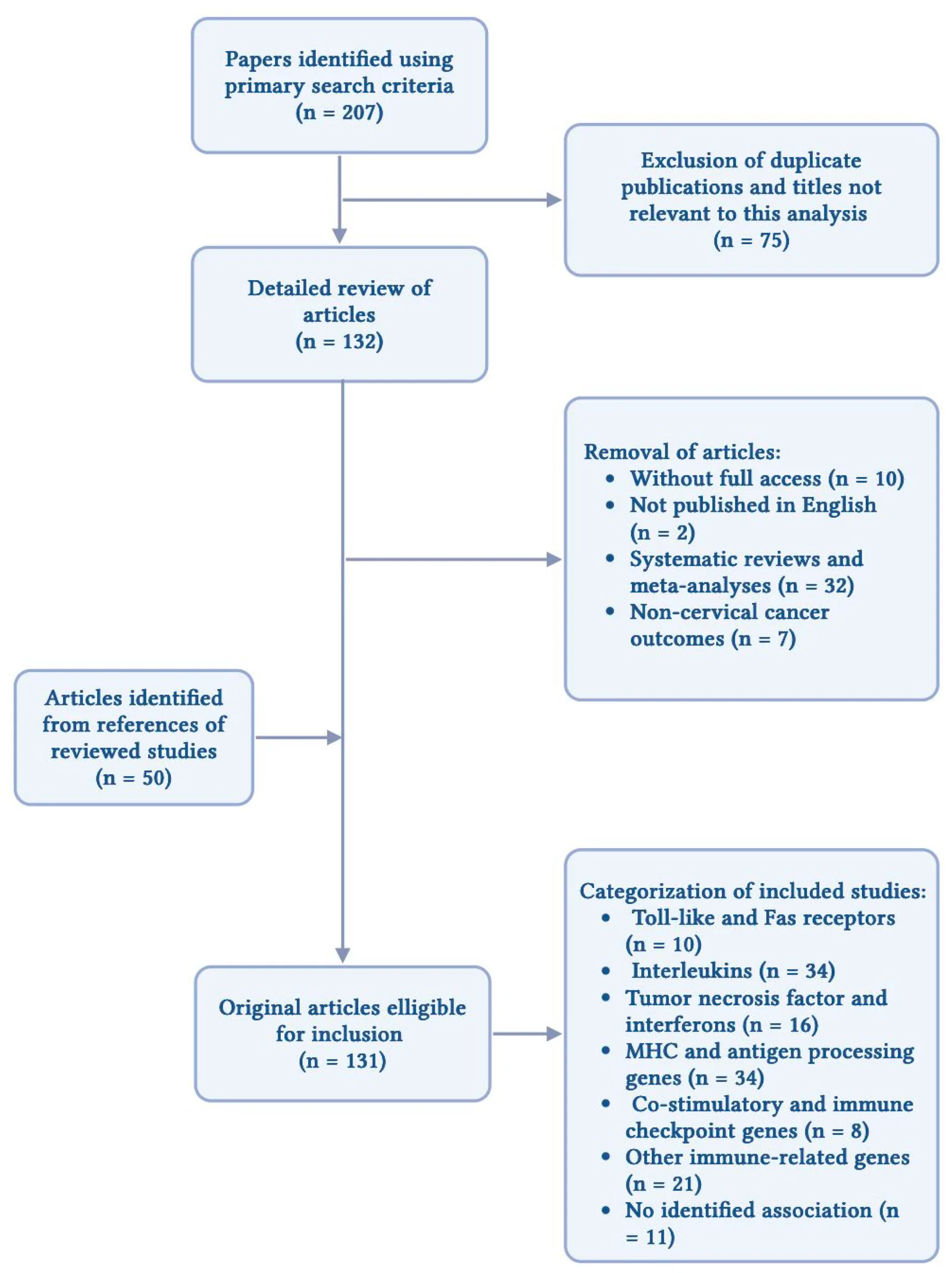
Strategy used to identify studies on immune-related genetic susceptibility to cervical cancer.

The detailed results of the literature search strategy are presented in Figure 1. The structured search was conducted to identify studies investigating immune-related genetic susceptibility factors associated with CxCa risk. The initial search identified 207 articles that met the primary criteria. During the first stage of screening, duplicate publications and articles not relevant to the topic were excluded, resulting in the removal of 75 papers. The remaining 132 articles underwent detailed evaluation.

At the eligibility stage, studies without full-text access, articles not published in English, systematic reviews, and meta-analyses were excluded. Additionally, studies focused on non-cervical cancer outcomes were removed.

Studies reporting both significant and non-significant associations between the analyzed immune response genes and CxCa susceptibility were included to provide a comprehensive overview of the available evidence. Given the heterogeneity of the included studies with respect to study design, investigated polymorphisms, and study populations, direct comparisons between studies are limited. An additional 50 articles were identified through screening the reference lists of the included studies, resulting in a total of 131 original articles that met the eligibility criteria and were included in the review.

The included studies were further categorized according to the type of immune-related gene analyzed. Since some studies investigated multiple immune-related pathways, three articles were classified into more than one group. Studies reporting no significant association between the analyzed genetic variants and CxCa risk were additionally considered as a separate category.

As a narrative review, this study was based on a literature search conducted in PubMed and was not pre-registered. A formal risk-of-bias or quality assessment was not performed, reflecting the narrative nature of the review. These aspects should be considered when interpreting the summarized evidence.

## 3. Candidate Immune Response Genes for Cervical Cancer Risk

**Figure 2:**
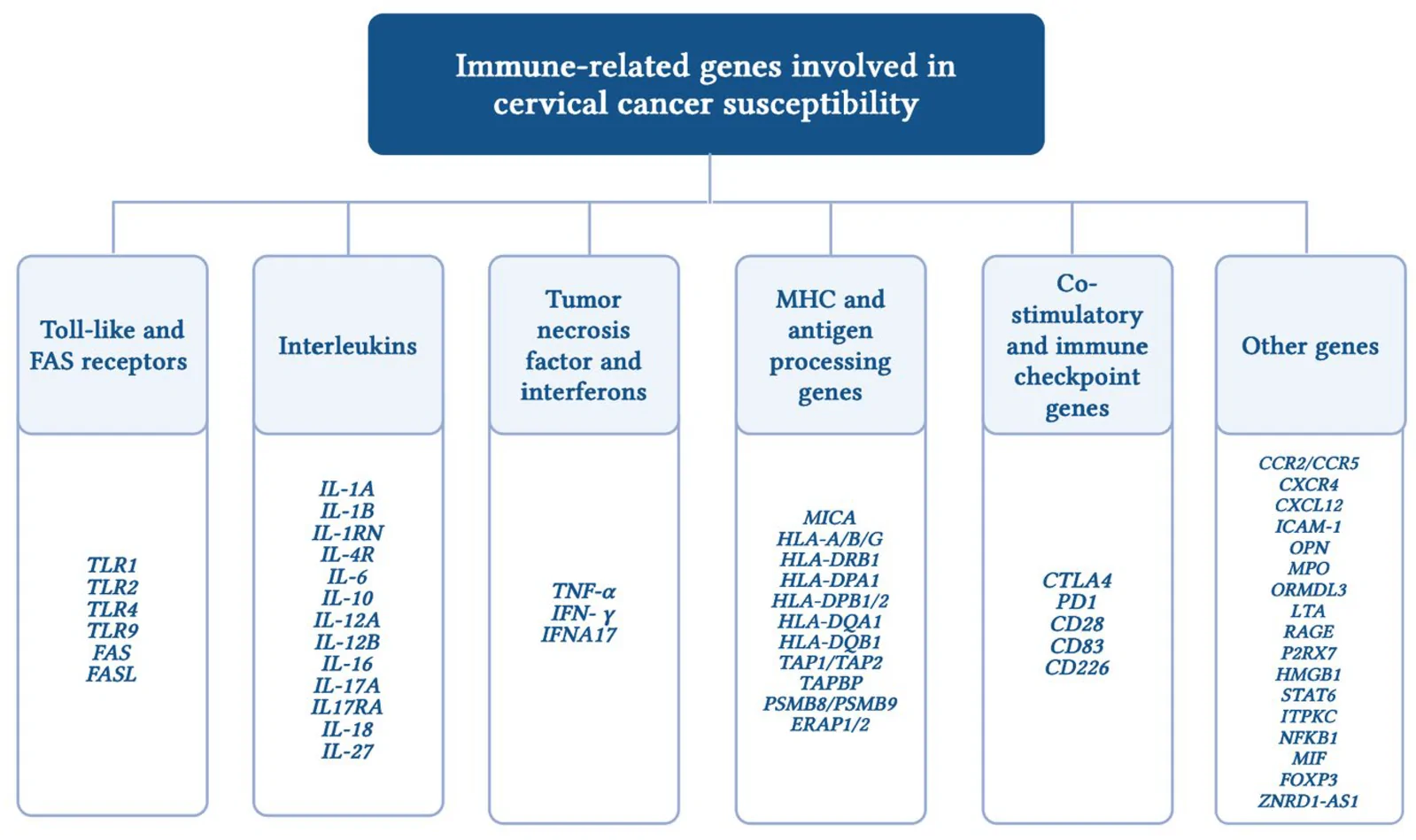
Immune-related gene groups with key examples of genes relevant to cervical cancer.

Recent improvements in cervical cancer research have increasingly emphasized the importance of genetic factors in modulating individual susceptibility to both HPV-related and HPV-independent pathways of carcinogenesis [10]. Although HPV remains the major cause of CxCa, the presence of certain polymorphisms can significantly influence individual risk, meaning that infection may not inevitably lead to the development of the disease [12]. Approximately 27–36% of the liability of CxCa is determined by heritability via genetic variants [13,14]. Current studies are focused on various groups of genes, with the strongest body of evidence pointing to genes related to the immune response. They constitute one of the most extensively studied groups, as both innate and adaptive responses are closely linked with HPV infection and the immune activity against the virus plays a crucial role in CxCa pathogenesis [15]. The most important groups of these immune-related genes linked to CxCa risk are summarized in Figure 2.

As illustrated above, the most widely investigated are genes encoding Toll-like receptors (TLRs), interleukins (ILs), tumor necrosis factor (TNF), interferons (IFNs), human leukocyte antigens (HLA), as well as molecules involved in apoptosis, such as the FAS/FASL system, and immune cell surface markers, including cluster of differentiation (CD) proteins.

TLRs are innate immune receptors that recognize specific molecular patterns of pathogenic microorganisms. Their activation initiates signalling pathways leading to a rapid inflammatory response [16,17].

The FAS/FASL system represents a receptor–ligand pair responsible for apoptosis (programmed cell death). The binding of FAS ligand (FASL) to its receptor (FAS) triggers intercellular signaling cascades that lead to the controlled elimination of cells [18,19].

ILs are a group of cytokines, or signaling proteins, between immune cells. They are responsible for regulating inflammation and mediating communication between leukocytes [20].

TNFa is a proinflammatory cytokine that plays a key role in immune and inflammatory processes. It contributes to the amplification of inflammatory responses, and under certain conditions, may also induce cell death [19].

IFNs constitute a group of signaling proteins produced primarily in response to viral stimuli. They inhibit pathogen replication and enhance the activation of other immune cells, thereby contributing to the regulation of immune responses [21].

HLA are cell surface molecules responsible for presenting antigenic peptides to lymphocytes. They are essential for immune recognition, particularly in distinguishing between self and non-self-components [22,23,24].

Additionally, CD molecules represent a set of surface markers expressed on immune cells (e.g., CD4, CD8). They are widely used to identify specific cell populations and also participate in the regulation of immune responses [25].

### 3.1. Toll-Like and Fas Receptors

As mentioned above, polymorphisms in genes encoding Toll-like receptors are being investigated for their potential impact on cervical cancer risk. Toll-like receptors are transmembrane proteins that detect microbial antigens and signals from damaged tissues, initiating cytokine responses linking innate and adaptive immunity [26,27].

For instance, TLR1 forms a heterodimer with TLR2 to recognize bacterial triacylated lipoproteins [28]. A study conducted by Moura et al. showed that rs5833095 SNP in *TLR1* was associated with an increased risk of CxCa and may affect the structure and stability of the encoded protein [29]. Additionally, the allelic combination CAG (rs4833095/rs4986790/rs187084) was found to further increase the risk of CxCa [29]. Similarly, a comparable effect was observed for the *TLR2* (−196 to −174) del variant in North Indian women [30].

TLR4 likewise plays a significant role in the recognition of lipopolysaccharide molecules from Gram-negative bacteria [31]. Recent studies have found an association between *TLR4* SNPs and the development of CxCa across different populations. In Chinese populations, rs10116253 (C), rs1927911 (T) and rs10759931(G) were reported to be linked to a reduced risk of CxCa, whereas rs7873784 (G) was associated with an increased risk [32,33]. In contrast, findings from the Indian population indicate that rs1927911, rs11536889, rs4986790 and rs4986791 were related to a higher disease risk [30,34]. Haplotype analyses further supported the importance of *TLR4*, showing that the *TLR4* haplotype GTAC was associated with an increased risk of CxCa, while the haplotype GCAG was linked to a decreased risk [34]. In the context of HPV infection, genetic variation in *TLR4* was also shown to influence the high-risk HPV co-infections. Specifically, the *TLR4* rs10759931 SNP appeared to exert a protective effect against multiple hrHPV infections, whereas the *TLR4* haplotype ACAC was associated with an increased risk, which may also have important implications for CxCa development [35].

A similar pattern is observed for TLR9, which recognises unmethylated CpG sequences present in viral genomes such as HPV16 [36]. Various studies have shown that the *TLR9* rs187084, located in the LD block with rs352140, was associated with an increased risk of CxCa [34,37]. rs352140 likewise increased risk in Chinese and Polish cohorts, with the GA + AA genotypes conferring the highest susceptibility in HPV-infected individuals [32,38,39]. The *TLR9* haplotype GATC was also linked to an elevated risk [34], whereas rs187084, rs352140 and rs352139 were associated with a reduced likelihood of high HPV16 copy number, suggesting a modulatory effect on viral load [35].

In addition to TLRs, other immune regulators have been linked to CxCa susceptibility, including the FAS receptor and its FASL ligand. Upon interaction with FASL, the cell-surface FAS receptor activates apoptotic signaling, enabling the removal of virus-infected cells [40]. rs1800682 (c.-670A>G) in the promotor region of the *FAS* gene has been found to be associated with cervical carcinogenesis in several populations [41,42]. Conversely, rs2234767 (c.-1377A>G) is not individually linked to cervical neoplasia, but it amplifies the effect of rs1800682 [43].

Regarding *FASL*, the rs763110 (c.-844T>C) has been reported to confer a threefold increased risk of CxCa in individuals with the CC genotype in a Chinese population. This elevated risk may be explained by higher FASL expression associated with the C allele, which can enable tumor cells to evade immune surveillance [44]. Moreover, evidence suggests that the *FAS* rs2234767A-rs1800682A haplotype, in combination with the *FASL* rs763110 C allele, is linked to an increased susceptibility to CxCa [43]. The reported effects of polymorphisms in the receptor-encoding genes are summarized in Supplementary Table S1.

### 3.2. Interleukins

Another widely examined group within immune response genes comprises interleukins. These cytokines are produced by various cell types and control immune and inflammatory processes by modulating the activation, differentiation, proliferation, and communication of immune cells. Moreover, they exhibit both pro-inflammatory and anti-inflammatory properties, acting through binding to specific cell-surface receptors [20]. A large number of studies have investigated the role of interleukin genes in CxCa risk, most frequently focusing on *IL-1B*, *IL-1RN*, *IL-6, IL-10*, *IL-12B* and *IL-17A*. Among the interleukins listed above, IL-1β, IL-6, IL-12B and IL-17A are primarily involved in pro-inflammatory immune mechanisms [45,46,47,48]. In contrast, IL-1RN and IL-10 are mainly associated with anti-inflammatory functions, restraining excessive immune activity [49,50,51].

Regarding the *IL-1B* gene, the most consistently reported SNP in the context of CxCa risk is rs16944 (c.-511C>T/c.-511A>G) located in the promotor region. This polymorphism has repeatedly been associated with an increased susceptibility to CxCa, with the TT and GG genotypes showing an increased risk in Chinese, Bangladeshi, Indian, and Korean cohorts [52,53,54,55]. Additionally, rs1143627-AA has been reported to confer a nearly two-fold increased risk, whereas rs3136558 and rs114360 have been identified as protective variants, each linked to a significantly reduced likelihood of CxCa development [52]. Notably, haplotype analysis revealed that the TGA haplotype (rs1143630/rs1143627/rs16944) significantly decreases disease susceptibility, suggesting that the combined effects of SNPs may modulate CxCa risk more effectively [52].

Several studies have highlighted the role of *IL-6* polymorphisms, particularly rs1800795 (c.-174G>C), rs2069837, and rs1800797 (c.-572G>A), in the susceptibility to CxCa in different populations. Research findings indicate that carriers of the C allele and CC genotype of rs1800795 have an elevated risk of CxCa [56,57,58,59,60,61,62]. In Lithuania, the CC genotype was observed more frequently among HPV-positive individuals compared to the HPV-negative group [56]. Moreover, the CC genotype of rs1800795 is associated with advanced International Federation of Gynecology and Obstetrics (FIGO) stage III-IV in Chinese population, while the GC genotype increases the risk of stage I tumors in Indian patients [58,61]. A study conducted by Pu et al. also showed a significantly higher risk of CxCa in carriers of the G allele of rs2069837, and GDMR analysis revealed a significant gene–gene interaction between rs1800795 and rs2069837 (*p* = 0.0010) [59]. In a separate two-stage case-control study in Eastern Chinese women, rs2069837 was linked to an increased CxCa risk, with AG/GG carriers showing higher IL-6 expression and evidence of interactions with reproductive factors [62]. Regarding the rs1800797 polymorphism, the A allele and AA or AG genotypes were found to be associated with an increased CxCa susceptibility in the Bangladeshi population [53], whereas the same genotypes were linked to a decreased risk among Swedish women [63]. In addition, among the Lithuanian population, the G allele was more frequent in individuals with HPV16/18 (*p* = 0.0045), while women with the AA genotype of rs1800797 were less likely to be infected with these HPV types [56].

Polymorphisms within the *IL-12B* have also been linked to CxCa susceptibility. For rs3212227 (c.1188A>C), studies from Poland and India indicate that the AC and CC genotypes confer an increased risk of developing disease [64,65]. In the Indian population, this effect was further amplified among passive smokers, where the presence of these genotypes increased risk more than 2.8-fold compared with nonsmokers carrying the AA genotype [65]. Findings from China also support the involvement of rs3212227, showing that AC and CC genotypes contribute to higher risk, and a gene–gene interaction with *IL-12A* rs568408 further enhances susceptibility [66]. A study conducted by Han et al. identified additional *IL-12B* variants linked to CxCa risk in the Korean population [67]. The GG genotype of the IVS2-912G/A polymorphism was associated with an elevated risk. Moreover, haplotypes including IVS2-912G:IVS4 + 314A, IVS2-912G:IVS4 + 314A:3′UTR + 1188A, and IVS2-912G:IVS4 + 314A:3′UTR + 1188C showed significant associations with an increased susceptibility [67].

The *IL-17A* rs3748067 CT genotype was associated with an increased CxCa risk in Bangladeshi patients, both in codominant and overdominant models [68]. Studies from China emphasize the involvement of rs2275913, where carriers of the GA or AA exhibit markedly increased risk compared with GG homozygotes [69,70,71]. This variant was also correlated with clinicopathological features, including positive peritumor intravascular cancer emboli and a high clinical stage of CxCa [71]. Importantly, the risk conferred by rs2275913 was particularly higher among women carrying HPV16 or HPV18, highlighting a significant gene–HPV interaction [69,70].

The *IL1RN* gene, which encodes the interleukin-1 receptor antagonist (IL1RA), plays a key role in modulating the host immune response [72]. It contains an 86-bp variable number of tandem repeats (VNTR) polymorphism in intron 2, with five known alleles. The most common are allele 1 (four repeats, 420 bp) and allele 2 (two repeats, 240 bp) [73]. This polymorphism has been implicated in CxCa susceptibility. In the Portuguese population, the 2/2 genotype was significantly more frequent among high-grade squamous intraepithelial lesion + invasive cervical cancer (HSIL + ICC) cases and was associated with nearly a two-fold increase in disease risk. Additionally, women homozygous for allele 2 also developed disease at a younger age compared to non-2/2 individuals [74]. Data from India show that the 2/2 genotype was associated with more advanced disease, particularly stage III of CxCa [54]. There is also evidence of a strong protective association for the 1/2 genotype, which markedly reduced the likelihood of HPV18 infection. A similar protective effect was seen for the combined 1/2 + 2/2 and genotypes, both in relation to HPV19 and to co-infection with HPV16 and HPV18 [75].

Regarding *IL-10*, four polymorphisms (rs1800896, rs1800872, rs1800871, rs3024490) in promoter and intronic regions displayed notable associations with CxCa risk, although the results of studies vary across populations. For rs1800896 (c.-1082A>G), research indicates a population-dependent effect. The GG genotype was found to be associated with a reduced HSIL and cervical cancer risk in Brazilian women [76], whereas in India and Bangladesh it was linked to an increased likelihood of CxCa [77,78]. A study conducted among Japanese women found that the carrier frequency of the *IL-10* rs1800896 G allele, associated with higher IL-10 production, increased with disease severity [79]. In addition, haplotype analysis showed that the combination of rs1800896 and rs1800872 in the form of the AA haplotype significantly increased the risk, while the CA haplotype lowered the risk [78]. Similarly, the effect of rs1800872 (c.-592 C>A) varies by population. In Bangladesh and Tunisia, the AA genotype was associated with an elevated risk of CxCa [78,80], while in North Macedonia, this genotype appeared to confer a protective effect [81]. A study conducted by Du et al. showed that the combination of *IL-10* rs1800872 CA or AA genotypes with the *TNF* rs1800629 variant was linked to an increased disease severity [82]. Evidence from the Netherlands also suggests that individuals heterozygous for the A allele of *IL-10* rs1800872 had a higher risk of CIN II-III and squamous cell carcinoma of the cervix [83]. Moreover, homozygous TT carriers of *IL-10* rs1800871 (c.-819C>T) exhibited a significantly higher risk of CxCa compared with CC homozygous [80]. A similar effect was observed in the Chinese population, where individuals with the CT + TT genotypes also showed increased susceptibility [84]. Finally, the rs3024490 variant appears relevant, as the TT genotype has been associated with an increased risk of developing disease among Tunisians [80]. The same study found that the haplotypes GTCCA and TGATG, containing polymorphisms in *IL-10* associated with CxCa risk, were less frequent in cases compared to controls, suggesting a protective effect [80].

There is also evidence suggesting that other polymorphisms in interleukin-related genes may influence susceptibility to CxCa. Other variants associated with an increased risk include rs719250 (*IL1R2*), rs3218896 (*IL1R2*), rs1801275 (*IL-4R*), rs568408 (*IL-12A*), rs1131445 (*IL-16*), rs879576 (*IL17RA*), and rs187238 (*IL-18*) [53,66,85,86,87,88]. Conversely, polymorphisms in *IL-27* (rs17855750) and in *IL-1A* (rs3783553) appear to confer a protective effect [89,90,91]. Additionally, the variant in *IL-4R* (rs1805010) has shown inconsistent or population-dependent associations, where risk effects vary across populations [81,92]. These findings, presented in Supplementary Table S2, indicate that both pro- and anti-inflammatory interleukins may play a role in modulating individual susceptibility to CxCa.

### 3.3. Tumor Necrosis Factor and Interferons

Tumor necrosis factor alpha and interferons play a key role in antiviral immunity and inflammatory signaling, and polymorphisms within genes encoding these cytokines have also been implicated in the risk of developing CxCa.

TNF-α is a pro-inflammatory cytokine involved in cell proliferation and differentiation, produced primarily by monocytes and macrophages [93]. Beyond its physiological function, TNF-α is frequently overexpressed in various malignancies and has been implicated in processes critical to carcinogenesis [94]. The polymorphisms located mainly in the promoter region of the *TNF-α* gene are suspected to contribute to various diseases, such as CxCa [95]. However, the associations remain inconclusive across populations. The most extensively studied variant, rs1800629 (c.-308G>A), has been repeatedly linked to the risk of developing CxCa among individuals from diverse populations. The presence of the A allele or GA/AA genotypes has mainly been associated with higher CxCa risk [82,96,97,98,99,100,101,102,103,104]. Only in one study conducted by Yang et al. was the rs1800629 A allele found to be a protective factor for CxCa [94]. Additionally, this variant has been associated with advanced tumor stage (III and IV) in the Polish population [99]. There is also evidence that rs1800629 may enhance susceptibility to high-risk HPV infection, particularly HPV16 and HPV58 [102,105]. Similar patterns have been observed for rs361525 (c.-238G>A), where both risk-enhancing and protective associations have been reported. Depending on the study and the analyzed population, this variant has been associated either with an increased susceptibility to cervical cancer or with a reduced risk [82,94,98]. Regarding rs1799724 (c.-857C>T), studies conducted in Bangladeshi and Chinese populations indicate that the presence of the TT genotype or the T allele was linked to a higher likelihood of developing CxCa [96,106]. In addition, the allele distribution of this variant differed significantly according to HPV infection status [106]. rs1799964 and rs1800630 and the *TNF-α-11* microsatellite polymorphism may also influence CxCa risk [98,107,108].

Interferon gamma (IFN-γ), a cytokine involved in the induction and modulation of immune responses, has been implicated in cervical carcinogenesis [109]. Research has mainly concentrated on the *IFNG rs*2430561 (c.874A>T) polymorphism. Studies conducted in China and India showed that the AA genotype, which is associated with lower IFN-γ production, was linked to an increased risk of CxCa [110,111]. In the Indian population, this genotype was additionally associated with advanced disease stages and showed a stronger effect among tobacco users. Evidence from Brazil further suggests that CxCa susceptibility may be modified by gene–gene interactions, as the combined presence of *IFNG* rs2430561 AA and *CD28* rs3116496 TT genotypes was more frequent among CxCa patients [112]. Beyond rs2430561, the CA-dinucleotide repeat polymorphism located in the first intron of *IFNG* has also been associated with CxCa risk. In the Taiwanese population, genotypes containing 12 or 14 CA repeats were more common in individuals with HSILs and showed an increasing significance in relation to disease severity. Furthermore, genotypes containing 13 or 18 repeats were more prevalent among controls and appeared to have a protective effect. These variants were additionally linked to HPV infection, indicating a possible involvement in susceptibility to specific HPV types 113.

Considering the role of interferons in cervical cancer risk, there is also evidence of an association with *IFNA17*. A study contributed by Kim et al. showed that the rs9298814 (c.551T>G) polymorphism in the *IFNA17* gene was associated with susceptibility to cervical squamous cell carcinoma (CSCC) [114]. The TT genotype was observed more frequently among the CSCC patients than in the group of women with normal cervical cytology [114].

Overall, these findings highlight the potential influence of *TNF-α*, *IFNG* and *IFNA17* polymorphisms on CxCa susceptibility, with detailed results summarized in Supplementary Table S3.

### 3.4. MHC and Antigen Processing Genes

The major histocompatibility complex (MHC) genes are essential in immune surveillance, enabling the processing and presentation of antigen peptides to T lymphocytes. In humans, the MHC comprises two main classes, MHC class I and MHC class II, which present antigens to CD8^+^ and CD4^+^, respectively [115]. These peptides are processed and transported within the cell by various components of the antigen-processing machinery (APM), which work in close association with MHC molecules [116]. There is evidence that both genes encoding MHC molecules and components of the AMP may influence susceptibility to CxCa.

Regarding MHC class I, polymorphism in the *MICA* gene, encoding a non-conventional MHC class I molecule expressed mainly on epithelial cells, showed significant association with CxCa [117]. The rs2844511 variant, located downstream of *MICA*, was linked to an increased risk of cervical disease, including invasive cervical cancer, high-grade dysplasia and low-grade dysplasia. Additionally, RNA analyses have revealed that MICA expression was upregulated in HPV16-positive samples [118]. Independent evidence from a nested case-control study in northern Sweden identified another *MICA*-related susceptibility signal, in which the allele T of rs2516448, in perfect linkage disequilibrium with the *MICA* A5.1 frameshift mutation, was associated with an increased CxCa risk [119].

There is also evidence that other MHC class I genes, including *HLA-A*, *HLA-B* and *HLA-G*, are associated with CxCa susceptibility. For *HLA-A*, several studies have been conducted on different populations, and they have shown a link. In a Chinese cohort, HLA-A*01:01 was more frequent in patients than controls, suggesting increased risk, whereas HLA-A*30:01 and HLA-A*33:03 were less frequent, indicating protective effects [120]. Moreover, a study conducted by Hosono et al. showed that the HLA-A*02:06 allele was significantly associated with a reduced CxCa risk in Japanese women, and this protective effect was independent of smoking status [121].

In the case of *HLA-B*, the rs1056429 polymorphism was linked to a lower risk of developing disease in a German population [122]. In a Swedish population, HLA-B*44, HLA-B*51 and HLA-B*57 were associated with markedly increased risks of CxCa among women infected with the HPV16 E6 variant L83V, conferring approximately a fivefold higher risk [123]. Further evidence from China demonstrates allele-specific effects within HLA-B*51:01:02 and HLA-B*51:01:01. The frequency of HLA-B*51:01:02 was significantly higher in patients with cervical squamous cell carcinoma (CSCC), while HLA-B*510101 appeared protective [124]. Additionally, haplotype analysis revealed a significant contribution of *HLA-A-HLA-B* haplotypes to CxCa susceptibility. The HLA-A*11-HLA-B*07 haplotype was strongly associated with an increased risk of disease. Conversely, the HLA-A*03-HLA-B*35 and HLA-A*11-HLA-B*35 haplotypes’ frequencies suggest a protective effect [125].

Recent research has demonstrated that the polymorphisms in the *HLA-G* gene are also associated with CxCa risk. Variants in the 3′ untranslated region of *HLA-G*, including rs1063320 (c.3142G>C) and the 14-bp deletion/insertion, were linked to an increased risk of CSCC. These associations were particularly pronounced in HPV16-positive cases, indicating potential interaction with HPV infection. In addition, the rs12722477 (c.1537A>C) polymorphism, when occurring together with the 14-bp deletion as a haplotype, was associated with an increased risk of CxCa in Taiwanese women [126].

For MHC class II, a larger body of research has demonstrated its impact on CxCa risk, particularly in the context of HPV infection. Key genes implicated include *HLA-DRB1*, *HLA-DPA1*, *HLA-DPB1*, *HLA-DPB2*, *HLA-DQA1* and *HLA-DQB1*.

There is extensive evidence that *HLA-DRB1* plays an important role in the risk of developing CxCa, and its influence varies depending on allele, haplotype, population, and HPV status. Several SNP-based studies have demonstrated significant associations. For instance, rs9357152 was linked to an increased risk of HPV16-positive CxCa and acted as an expression Quantitative Trait Loci (eQTL), lowering HLA-DRB1 expression in HPV-positive cervical tissues [127]. In contrast, the rs9272143 polymorphism consistently conferred a protective effect against CxCa in Bangladeshi and Swedish populations, with the protective C allele associated with higher HLA-DRB1 expression [119,128]. Allele-based studies also identified both risk-increasing and protective *HLA-DRB1* alleles. An increased risk of CxCa was associated with several *HLA-DRB1* alleles, including DRB1*07, DRB1*04, DRB1*15, DRB1*10:01 and DRB1*11:01 [63,125,129,130,131,132,133,134]. On the contrary, reduced risk was observed for DRB1*13:01, DRB1*13:02:01, DRB1*04:06, DRB1*10, DRB1*15:01, and DRB1*03:01 alleles [63,125,132,135,136].

A study conducted by Jiang et al. showed that the rs3077 polymorphism in *HLA-DPA1*, a gene encoding the DP alpha chain of the MHC class II complex and playing a key role antigen-presenting cell function [137], may be associated with an increased risk of CxCa. Carriers of the GA and AA genotypes were found to have an approximately 1.5-fold higher risk of cervical cancer compared with the reference genotype, indicating a significantly elevated susceptibility among variant allele carriers [138].

Several studies have demonstrated a significant impact of *HLA-DPB1* on CxCa. In a Taiwanese population, the DPB1*05:01 allele was associated with a reduced risk of CSCC, whereas the rs927753 polymorphism conferred increased risk. Stratified analyses have shown that, among HPV16-positive cases, the DPB1*05:01 allele remained protective, while DPB1*13:01 was associated with higher susceptibility [139]. Consistently, the rs927753 polymorphism has also been reported to be associated with an increased risk of CxCa in the Chinese population [138]. Further evidence supports the role of *HLA-DPB1* in CxCa, as the DPB1*13:01 and DPB1*02:02 alleles were significantly more frequent in patients than in controls, suggesting increased risk [140,141]. There is also evidence of a strong positive association between DPB1*13:01 and HPV16 E6 prototype-positive CxCa [142]. Additionally, a study conducted by Chen and Gyllesten identified a protective effect of the DPB1*04:02 allele, which was associated with a significantly reduced risk of CxCa among Swedish individuals [143].

Beyond *HLA-DPB1* alleles, polymorphisms at the neighbouring *HLA-DPB2* locus have also been implicated in CxCa susceptibility. *HLA-DPB2* is a non-coding pseudogene located within the HLA-DP region, and does not encode a functional antigen-presenting molecule [144]. A comparative study conducted in Sweden identified the *HLA-DPB2* rs3117027 polymorphism as the strongest association signal, with the A allele conferring higher risk of developing CxCa. Additionally, in silico analysis showed a strong correlation with rs3129294, suggesting a role in transcriptional regulation within the HLA-DP locus [143].

Evidence from multiple studies indicates that specific *HLA-DQA1* and *HLA-DQB1* variants may influence CxCa risk. Regarding *HLA-DQA1*, rs9272117 was linked to a lower risk of invasive cervical cancer and high-grade dysplasia, particularly in HPV16-positive cases in a German population [118]. Notably, a larger number of risk-associated alleles have been identified for *HLA-DQB1*. Specifically, several alleles were repeatedly linked to an increased susceptibility, including DQB1*03:01, DQB1*03:02:01, DQB1*03:03:02, DQB1*06:01:01, DQB1*02:01, DQB1*04:02 and DQB1*05:03:01, with particularly strong effects reported in HPV16-positive tumors [125,129,130,141,142,145,146,147]. Additionally, the rs6457617 polymorphism, where the A-allele-containing genotypes were more frequent in patients with uterine cervical cancer, further supports a risk-modifying role of *HLA-DQB1* [148]. In contrast, DQB1*03:19, DQB1*06:04:01, DQB1*06, DQB1*06:03, and DQB1*05:01 were associated with a reduced risk in several populations [129,130,136,145,146]. Remarkably, the DQB1*06:02 allele showed inconsistent effects, being linked to increased risk in some cohorts, while conferring protection in others [129,146,149].

Overall, the available evidence indicates that the most consistently replicated HLA associations involve variants within the *HLA-DRB1*, *HLA-DPB1*, and *HLA-DQB1* loci, highlighting the importance of antigen presentation in cervical cancer susceptibility. Among these, rs9272143 in *HLA-DRB1* has been repeatedly associated with a protective effect across independent populations [119,128], whereas *DPB113:01* has consistently been linked to increased susceptibility, particularly in HPV16-positive disease [138,139,140,141]. In contrast, several HLA alleles, including DQB1*06:02, have shown inconsistent associations across studies. Notably, DQB1*06:02 was associated with increased risk in two studies conducted in South African and Swedish populations [129,146], whereas a protective effect was observed in a Chinese cohort [149]. These discrepancies may reflect differences in ethnic-specific HLA allele frequencies and linkage disequilibrium patterns, variation in HPV genotype distribution, environmental and lifestyle factors, as well as differences in study design, sample size, and statistical power [150,151]. Given the extensive polymorphism of the HLA region and its strong population specificity, replication across ethnically diverse cohorts and functional studies are required to clarify the biological significance of these associations.

There is also evidence suggesting that numerous haplotypes of MHC class II influence susceptibility to CxCa [125,130,131,132,133,139,140,141,142,149,152]. However, given the sheer number and complexity of these haplotypes, a detailed description is beyond the scope of this review, and they are not individually discussed. All reported associations of MHC class I and II genes with CxCa are summarized in Supplementary Table S4.

As mentioned above, studies have shown that polymorphisms in APM genes contribute to CxCa susceptibility. In particular, *TAP1* and *TAP2*, which encode proteins that form a heterodimer essential for peptide transport and loading onto MHC class I molecules, have been reported to show associations with CxCa risk [153,154]. For *TAP1*, the T allele of rs41549617 and the G allele of rs1135216 were both associated with a decreased risk. Moreover, the alleles TAP1*02:01:01 and TAP1*03:01 were significantly linked to CxCa susceptibility [155]. Regarding *TAP2*, the G allele in rs241441 has been associated with a 1.2-fold increased risk of developing CxCa [155]. A study conducted by Mehta et al. showed that the rs4148876 in *TAP2* is also significantly associated with CxCa susceptibility in the Balinese population, with the minor allele conferring an increased risk of disease [156].

*TAPBP*, another gene involved in antigen processing and presentation, has also been implicated in CxCa susceptibility. The G allele of the SNP rs1059288, located in the 3′UTR of *TAPBP*, showed a significant association with increased disease risk. Additionally, this allele was linked to an enhanced m^6^A modification of TAPBP mediated by METTL14 and recognized by YTHDF2, leading to higher TAPBP expression in cervical tumor tissues. Functionally, the overexpression of TAPBP promoted cervical cancer cell proliferation and migration while reducing apoptosis and sensitivity to chemotherapy [157].

Evidence suggests that polymorphisms in the immunoproteasome subunit genes *PSMB8* and *PSMB9* are involved in susceptibility to cervical lesions and cervical cancer. Specifically, the TT and TG genotypes of the *PSMB8* rs2071543 SNP were associated with an increased risk of both cervical intraepithelial neoplasia (CIN) and CxCa. In parallel, the A allele as well as the AA and AG genotypes of the *PSMB9* rs17587, were linked to a higher risk of CxCa in a Chinese Han population [158].

Alterations in genes encoding endoplasmic reticulum aminopeptidases have also been shown to modulate the risk of CxCa. In *ERAP1*, several polymorphisms displayed significant differences in allelic and genotypic distributions between cases and controls. The rs26618 SNP, resulting in amino acid substitution (I276M), was associated with an increased risk, as individuals carrying the CC genotype showed a higher risk of developing CxCa [159]. In contrast, the minor alleles of rs27044, rs26653 and rs30187 were consistently linked to a reduced risk of CxCa in two independent studies [156,159]. For *ERAP2*, the G allele of the rs2287988 polymorphism showed a positive association with CxCa susceptibility [159]. Haplotype analyses further supported these findings, highlighting the impact of combinations of *ERAP1* and *ERAP2* variants on CxCa risk, with full haplotype details provided in Supplementary Table S5.

### 3.5. Co-Stimulatory and Immune Checkpoint Genes

Several molecules involved in T-cell regulation can either enhance or inhibit immune response. Polymorphisms in genes encoding co-stimulatory and immune checkpoint molecules may affect cervical cancer risk [160]. There is evidence that both variants promoting and those limiting T-cell activity can influence CxCa susceptibility. The impact of these molecules reflects the critical role of T-cell activity, where an appropriate balance between activation and inhibition is essential for effective immune defence [161].

CTLA4, expressed on regulatory T cells as well as CD4^+^ and CD8^+^ T cells, is one of the key molecules that suppress T-cell activity [162,163]. Research indicates that certain SNPs in *CTLA4* are linked to CxCa risk, with only a subset of its many genetic variants being studied extensively for their potential impact. Most studies on this gene have focused on the c.-318 C>T (rs5742909) polymorphism. The T allele and the CT genotype at this locus were consistently associated with an increased susceptibility to CxCa, particularly in HPV-related cases. In the Brazilian population, the T allele of rs5742909, as well as the TAG haplotype, including also rs231775 (c.49A>G) and rs3087243 (c.6230G>A), was linked to a higher risk of HPV, HSIL and CxCa [164]. Similarly, in a Taiwanese cohort, the CT genotype of rs5742909 was more frequent among patients with HPV16-positive CSCC compared with controls [165]. These results are also consistent with a study conducted by Pawlak et al., where the T allele and the combined TT + CT genotype of rs5742909 were associated with a higher risk of CSCC, especially in well-differentiated tumors [166]. Moreover, a *CTLA4* intronic (AT)n repeat polymorphism was overrepresented in patients, although it was not related to tumor grade [166]. An analysis in a Han Chinese population also demonstrated that the AA genotype of rs231775 contributed to an increased risk of CxCa, further supporting the role of *CTLA4* variants in cancer susceptibility [167].

Another gene encoding a molecule involved in the negative regulation of T-cell activity, *PD1*, has also been reported to influence susceptibility to CxCa. In a study of a Chinese cohort, the CT genotype of the PD-1.5 (rs2227981) polymorphism was associated with an increased CxCa risk, whereas the TT genotype and T allele alone were not significantly linked to disease risk [168].

Molecules that provide co-stimulatory signals to T cells have been investigated for their potential influence on disease risk. One of these molecules is CD28, which interacts with B7 ligands (CD80/CD86) on antigen-presenting cells to strengthen T-cell activation [169]. For instance, the rs3116496 polymorphism has been associated with increased susceptibility in the Chinese population, with carriers of at least one C allele showing higher risk [170]. Similarly, in Polish women, the CD28c.17+3C allele and CC + TC genotypes were more frequent among well-differentiated CSCC cases [166]. Additionally, individuals carrying the *CD28* rs3116496 TT genotype together with the *IFNG* rs2430561 AA genotype exhibited an increased risk in a Brazilian cohort [112].

Other genes encoding immune checkpoint molecules, including *CD83*, and *CD226*, have further been associated with CxCa risk in various populations. In the case of *CD83*, carriers of the CT and CC genotypes of rs750749 showed a 30–50% lower risk of CxCa compared with TT homozygotes [171]. Moreover, the rs727088 G allele in *CD226* was associated with an increased risk of CSCC in the Chinese population [172].

To sum up, detailed results of the studies investigating these co-stimulatory and inhibitory molecules are presented in Supplementary Table S6.

### 3.6. Other Immune-Related Genes

There is also evidence linking additional immune-related genes to CxCa risk. However, because these genes perform diverse and sometimes overlapping immune functions, they cannot be classified into any of the groups discussed above.

One such group of immune-related factors implicated in CxCa risk includes chemokines and their receptors, which play a central role in directing the movement of immune cells during immune and inflammatory responses [173]. The *CCR2* c.190G>A polymorphism has been associated with CxCa and HSIL, although the direction of this effect appears to differ between populations [92,174,175]. A study conducted in African women showed that the A allele of this variant was linked to an increased susceptibility to CxCa [175], whereas findings from a Swedish population suggest a protective effect of the same allele [92]. Regarding *CCR5*, an association between the Delta32 polymorphism and CxCa risk was observed in stage IB patients, where carriers of the *CCR5*+/delta32 genotype had more than a 4-fold risk of developing disease [176]. Furthermore, increasing evidence highlights the importance of the CXCL12/CXCR4 axis in CxCa risk. Polymorphisms in *CXCR4* and its ligand *CXCL12* have been linked to HPV infection and CxCa development. In Brazil, carriers of the *CXCR4* rs2228014 T allele showed a higher risk of HPV infection, SIL and CxCa. The *CXCL12* rs1801157 AA genotype was also associated with an increased susceptibility. Notably, the combined presence of both variants further amplified the risk, increasing the likelihood of cervical cancer by 4.2-fold [177]. Moreover, a study conducted by Yin et al. showed that the *CXCL12* rs266085 C allele was underrepresented in patients compared with controls, whereas the T allele appeared more commonly in those with the disease [106].

Genes encoding molecules related to cell adhesion may also influence the risk of developing CxCa. One of these is the gene encoding intercellular adhesion molecule-1 (ICAM-1), which is involved in promoting adhesion at the site of inflammation [178]. The rs5498 polymorphism of *ICAM-1* showed inconsistent results in different populations. The G allele was associated with a reduced risk of cervical adenocarcinoma (CAC) in a group of Chinese individuals, whereas in a Taiwanese population, GG homozygotes were linked to a higher likelihood of developing precancerous lesions and invasive cancer [179,180]. There is also evidence that carriers of CG and GG genotypes of the rs281432 SNP have an increased risk of developing CxCa [180]. Moreover, haplotypes analysis showed that the CTG haplotype (rs5498/rs3093030/rs281432) was protective in a Chinese population [179]. Studies have further found an association between osteopontin (OPN) and CxCa. This secreted protein indirectly supports cell adhesion by interacting with integrins, thereby promoting the attachment and migration of cells [181]. The rs17524488 and rs11730582 polymorphisms in *OPN* have been associated with CxCa risk. Individuals carrying the GG genotype of rs17524488 exhibited a higher likelihood of CxCa, whereas carriers of the TT and TC rs11730582 genotypes had a lower risk. Notably, these associations were observed only in HPV-positive women [182].

Similarly, stress response-related genes have been implicated in inflammatory processes and may contribute to the likelihood of developing CxCa. The myeloperoxidase (MPO) is a pro-oxidant factor released during oxidative stress and chronic inflammation that generates highly reactive oxidants capable of damaging DNA and other biomolecules, thereby creating conditions that may promote carcinogenesis [183]. In the case of CxCa, the rs2333227 (c.-463G>A) polymorphism located in the *MPO* promoter region has been linked to disease risk in different populations. A study conducted by Roszak et al. showed that the AA genotype was associated with a reduced risk of CxCa, particularly among women with a history of childbirth or non-smokers [184]. These findings contrast with results from Portugal, where the GG genotype, linked to higher ROS production, appeared protective, potentially by generating apoptosis-inducing radicals in transformed cells [185]. In a related context, *ORMDL3* encodes a transmembrane protein of the endoplasmic reticulum that probably participates in ER stress response and inflammatory pathways, and a recent study also indicates its potential involvement in CxCa development [186]. The G allele of rs8076131 in *ORMDL3* was significantly associated with an increased risk of CxCa in a Chinese Han population, with this association remaining significant in subgroups defined by later menarche age, higher parity, non-smoking status, and the absence of a family history of cancer [187].

In addition, polymorphisms in long non-coding RNAs (lncRNAs) may contribute to CaCx risk. *ZNRD1* is involved in transcriptional regulation, whereas its antisense lncRNA, *ZNRD1-AS1*, regulates ZNRD1 expression. The rs3757328A, rs6940552A, and rs9261204G variants in *ZNRD1-AS1*, identified as expression quantitative trait loci, were significantly associated with a reduced risk of CxCa in both dominant and additive models, with the protective effect increasing in a dose-dependent manner according to the number of variant alleles carried. Individuals carrying one to three variant alleles showed a decreased risk of CxCa compared with those carrying none (OR = 0.83, 95% Cl: 0.71–0.98), while carriers of four to six variant alleles demonstrated an even stronger protective effect (OR = 0.63, 95% Cl: 0.44–0.90). These findings suggest that SNPs within lncRNAs may influence cancer susceptibility by altering ZNRD1 expression and pathways involved in HPV-related carcinogenesis [188].

Recent research provides further evidence that other genes may also be associated with CxCa risk. Polymorphisms in *LTA* (rs2239704), *RAGE* (rs2070600), *P2RX7* (rs17525809), *HMGB1* (rs1412125, rs2249825), *ITPKC* (rs28493229), *NFKB1* (rs28362491) and *MIF* (rs5844572) were linked to a higher risk of developing disease [189,190,191,192,193,194,195]. In contrast, variants in *LTA* (rs1041981) and *FOXP3* (rs2294021, rs3761549) showed an association with a decreased likelihood of CxCa [196,197]. Additionally, the TT genotype of *TGFB1* rs1800496 polymorphism appeared to confer a marginal protection in stage I disease, whereas in more advanced stages it was linked to an increased risk [198]. Detailed results for all genes mentioned are presented in Supplementary Table S7.

### 3.7. Investigations Reporting No Significant Association with Cervical Cancer Risk

While certain immune response gene polymorphisms show well-replicated associations with CxCa susceptibility, several studies examining other variants have failed to confirm such links. These investigations included polymorphisms selected based on their potential involvement in immune regulation and inflammatory processes. However, despite their biological relevance, the analyzed variants did not demonstrate a statistically significant association with CxCa risk in the respective study populations. Therefore, only those studies in which no significant link to CxCa susceptibility was found are discussed below.

One study investigating Toll-like receptor gene polymorphisms reported no evidence of a link between the *TLR3* rs3775290 and *TLR9* rs352140 polymorphisms and CxCa risk in a North Indian population. Marginal effects were observed in selected subgroup analyses; however, these findings were not considered conclusive [199]. Data on *TLR9* rs352140 have varied across populations, with several studies reporting an increased CxCa risk, whereas no effect was found in a North Indian cohort [32,38,39].

Among genes encoding interleukins, studies investigating selected candidate polymorphisms reported no significant associations with CxCa susceptibility in certain populations. For example, no significant associations were identified for IL10 (rs1800896) or IL1B (rs16944) polymorphisms in a Croatian population [200], contrasting with findings from Brazilian, Bangladeshi, and Chinese cohorts [52,53,54,55,76,77,78,79]. Similarly, an Argentine case–control study found no association between IL10 rs1800896 and either HPV infection or CxCa risk [201]. In Taiwanese women, polymorphisms in IL18 (c.-1298T>C, c.-607C>A, c.-380C>G, c.-137G>C, and c.105A>C) were not associated with CSCC or HPV16-positive disease [202]. These findings reflect population-specific results for selected candidate variants and should be distinguished from genome-wide association studies identifying multiple MHC/HLA and non-HLA susceptibility signals.

Regarding tumor necrosis factor, two studies investigating *TNF-α* polymorphisms did not identify a link between the analyzed variants and CxCa risk. In women from the Republic of North Macedonia, the *TNF-α* rs361525 and rs1800629 polymorphisms showed no differences in genotype or allele distributions between patients with HPV-associated CIN/CxCa and healthy controls [203]. Likewise, a study conducted in Argentina evaluating the same variants in *TNF-α* reported no association with HPV infection or CxCa development. Although a potential protective effect was suggested for heterozygous carriers of the *TNF-α* rs361525, the evidence was insufficient to support a confirmed effect on CxCa risk [201]. Notably, the contribution of both *TNF-α* variants remains unclear, with studies reporting heterogeneous findings ranging from a lack of evidence for an effect to potential protective and risk-enhancing effects [82,94,96,97,98,99,100,101,102,103,104].

Within the MHC region, the analysis of *MICA* alleles in a northeastern Thai population revealed no differences between CxCa cases and controls without disease. Similarly, *MICA* alleles were not linked to HPV infection status, HPV type or disease stage. Although the c.638T>C and c.752G>A substitutions initially appeared to be associated with CxCa risk, these findings were not retained after correction for multiple comparisons [204].

Other immune response genes have likewise yielded negative findings. In a Brazilian population, AG and GG genotypes of the *TGFBR2* rs3087465 polymorphism initially appeared to increase CxCa risk. However, this effect was no longer evident after adjustment for potential confounding factors. The same study also found no evidence of a link between *FOXP3* rs3761548 and CxCa susceptibility [205]. Similarly, the *CCR5* rs333 variant was not related to HPV infection or cervical lesion development [206], while the *NKG2C* deletion polymorphism showed no effect on HPV infection or progression from low-grade squamous intraepithelial lesions (LSIL) to HSIL [207]. Furthermore, analysis of the *NFKB1* rs28362491 and *NFKBIA* rs696 variants did not support their involvement in HPV infection, cervical lesion development or disease progression [208]. Detailed results are summarized in Supplementary Table S8.

Evidence from large-scale genetic analyses further supports the limited role of immune-related genetic variants in CxCa risk. A recent Mendelian randomization study based on GWAS summary statistics from the UK Biobank, including more than 475,000 individuals of European ancestry and approximately 24.2 million genetic variants, found no substantial associations between genetically predicted immune cell characteristics and CxCa susceptibility. Across multiple MR approaches, the estimated effects remained consistently negligible, with an odds ratio close to 1.0, supporting the absence of a major independent genetic contribution to disease risk [209].

Overall, the studies summarized in this section highlight the challenges in identifying reproducible genetic determinants of CxCa susceptibility. The lack of confirmed effects for numerous investigated variants may reflect differences in genetic background, HPV-related factors, and environmental exposures [11]. These findings emphasize the importance of negative results in assessing genetic association and the need for further large-scale studies to define their role in CxCa risk [210].

### 3.8. Summary of the Most Consistently Replicated Associations

Although not all investigated immune response gene polymorphisms have demonstrated significant associations with CxCa risk, a subset of variants has shown consistent replication across independent populations and studies. In this review, “consistently replicated” was operationally defined as an association reported in at least two independent studies or cohorts. This criterion represents a modest threshold for replication and should not be interpreted as evidence equivalent to meta-analytic-level confidence. Table 1 provides an overview of the polymorphisms with the strongest support in the literature, together with the populations in which these findings were reported and their potential biological relevance. Consistency was determined based on repeated observations across independent cohorts and/or different ethnic groups. For publications originating from the same or overlapping research groups, the underlying study populations were additionally examined to assess whether the reported findings represented independent cohorts. In particular, the study populations reported by Huang et al. (2015) [90] and Pu et al. (2014) [91] for the *IL1A* rs3783553 polymorphisms, as well as those described in the two populations by Wu et al. (2007) [140,142] concerning HLA-DQB1*06:01:01, could not be confirmed as fully independent based on the information provided in the respective articles. Consequently, these publications were not considered to represent independent replication. The identified variants were predominantly located in genes involved in innate immune recognition, T-cell activation, antigen presentation, and cytokine signaling.

This observed consistency among studies may suggest that these variants are likely to be functionally relevant in key immune pathways involved in CxCa pathogenesis. However, heterogeneity in study design, ethnic differences, environmental exposures, and limited statistical power may also contribute to variability in the replication of other reported associations [11].

### 3.9. Evidence from Genome-Wide Association Studies and Candidate Gene Studies

Studies on the genetic susceptibility to CxCa have so far relied primarily on two approaches: genome-wide association studies (GWAS) and candidate gene studies. The vast majority of publications included in this review are candidate gene studies, which analyzed previously identified genes associated with immune function. This has led to the identification of numerous polymorphisms in genes involved in antiviral response, inflammation regulation, antigen presentation, and lymphocyte activation, including *TLR*, *IL*, *TNF*, *IFNG*, *CTLA4*, *PDCD1*, *TAP*, *ERAP*, and *FAS/FASL* (e.g., [29,30,32,33,34,35,36,108,110,114]. However, the results of these studies often differed between populations, and many of the observed associations were not unequivocally confirmed in subsequent analyses.

A different approach is used by GWAS studies, which analyze genetic variation across the entire genome without prior identification of potentially disease-related genes. Previous GWAS studies of cervical cancer have shown that the strongest and most reproducible genetic signals are located within the major histocompatibility complex (MHC) region on chromosome 6 [118,126,127,128,138,139,140,141,142,143]. Among the genes associated with immune response, the most significant were *HLA-DPA1*, particularly the rs3077 variant [138], and the *IL10* haplotype encompassing the rs1800872/rs1800896 polymorphisms [78], which reached the threshold of genomic significance. Furthermore, GWAS studies and subsequent fine mapping analyses confirmed the involvement of the *HLA-DRB1*, *HLA-DPB1*, *HLA-DPB2*, *HLA-DQB1*, and *MICA* genes [119,127,128,138,143,145], emphasizing the key role of antigen presentation in the immune response against HPV infection. Recent GWAS meta-analyses also indicated the involvement of the *GSDMB* gene, whose product participates in the regulation of pyroptosis and the inflammatory response, thus expanding the list of immune system genes associated with susceptibility to CxCa development [211].

Overall, current evidence indicates that while candidate gene studies have implicated a broad spectrum of immune response genes in CxCa susceptibility, only a limited number of loci have been validated by GWAS. The strongest and most consistently replicated associations remain confined to the HLA region, highlighting antigen presentation as the key genetically determined immune mechanism underlying CxCa development. Future large-scale GWAS involving diverse populations and functional validation studies will be necessary to determine whether additional immune-related loci contribute to disease risk beyond the HLA region.

### 3.10. Immune Response Gene Polymorphisms and HPV-Related Outcomes

The significance of immune response gene polymorphisms in the development of CxCa may be partly linked to their influence on the course of HPV infection [11]. Although exposure to the virus is common, most infections are spontaneously cleared; the development of precancerous lesions and cancer is primarily driven by persistent infection with high-risk HPV types [212]. Genetic variations affecting viral recognition and the efficacy of the antiviral response may therefore determine whether the infection is cleared or persists, creating conditions conducive to the subsequent stages of carcinogenesis [156].

Some of the studies included in this review went beyond assessing the general risk of CxCa and also analyzed the variants in relation to HPV infection. The scope of these analyses varied, covering factors such as the presence of HPV or HR-HPV infection, infection with a specific viral type, HPV16 viral load, co-infection with multiple high-risk types, and the development of CIN or HSIL lesions in HPV-infected women. In other studies, the endpoint was HPV-positive CxCa, sometimes restricted to a specific histological type or viral variant.

The observed associations involve genes participating in various stages of the immune response. Variants of *TLR4* and *TLR9* have been linked primarily to susceptibility to HPV infection, a finding that may stem from the role the receptors they encode play in the early recognition of the pathogen [34,39]. Meanwhile, polymorphisms in genes encoding cytokines and their receptors—such as *IL4R*, *IL6*, *IL10*, *IL17A*, and *TNF-α*—have been analyzed in relation to both the presence of infection and the development of HPV-positive intraepithelial lesions or CxCa [56,69,70,79,81,87,102]. These results indicate that the significance of specific variants may manifest at different stages of the response to HPV—ranging from the initial control of the infection to the regulation of persistent inflammation.

A particularly numerous groups of associations comprises those involving *HLA* genes. HPV infection and the occurrence of HPV-positive CxCa have been associated with variants or alleles of *HLA-B*, *HLA-G*, *HLA-DRB1*, *HLA-DPB1*, *HLA-DQA1*, and *HLA-DQB1* [118,123,126,127,129,132,134,139,140,142,145,148,149]. Depending on the variant studied, both increased and decreased risk were observed. The diverse direction of these associations may reflect differences in the ability of individual HLA molecules to present HPV antigens and activate a specific lymphocyte response. Associations with HPV-related endpoints have also been reported for genes involved in the regulation of lymphocyte activity, immune cell migration, and inflammatory response, including *MICA*, *CTLA4*, *CXCR4*, *CXCL12*, *OPN*, *RAGE*, *P2RX7*, and *FOXP3* [118,164,165,177,182,189,190,197].

However, the interpretation of these observations is hampered by significant heterogeneity across studies. Individual analyses were conducted in different populations and clinical groups, using different HPV detection and genotyping methods. Differences also applied to the types and variants of the virus analyzed. Crucially, however, no uniform endpoint was used; some studies assessed the mere presence of infection, others assessed the presence of CIN or HSIL lesions in HPV-positive women, and others assessed the development of invasive HPV-related cancer. In some analyses, HPV status was determined only once, making it impossible to determine whether the detected infection was transient or persistent.

Based on the currently available evidence, it remains difficult to clearly assign the described variants to specific stages of the natural history of HPV infection. The association observed for HPV-positive CxCa may result from increased susceptibility to infection, the reduced efficacy of its elimination, or the impact of a given variant on the progression of existing lesions [11]. It is also difficult to clearly distinguish variants that promote HPV persistence from those that are primarily important for cancer progression.

These methodological differences substantially limit direct comparisons between studies and may partly explain the inconsistent associations reported for several variants. This subsection includes only additional analyses of HPV derived from studies and relating to the gene variants discussed in earlier sections of the paper. It does not include all variants analyzed in the cited publications, nor does it constitute a separate, comprehensive review of the genetic determinants of HPV infection. A detailed summary of the variants considered, the study populations, the endpoints used, and the directions of the observed associations is presented in Table 2.

## 4. Discussion, Future Perspectives and Conclusions

### 4.1. Biological and Clinical Implications

This review summarizes the current data on the contribution of immune response genes to CxCa susceptibility. While persistent infection with high-risk human papillomavirus remains the primary etiological factor and the stronger predictor of CxCa development, accumulating evidence indicates that genetic factors, particularly those involved in immune regulation, significantly modulate the risk of disease susceptibility and progression [213]. GWAS and candidate gene studies have been essential in identifying CxCa susceptibility loci in the general population, highlighting the crucial role of innate and adaptive immune pathways in developing disease [151]. Although a large number of variants within immune response genes have been identified as potentially associated with CxCa risk, only haplotype CA (rs1800872/rs1800896) of *IL-10* and rs3077 of *HLA-DPA1* have reached the genome-wide significance threshold (2.42 × 10^−8^ and 5.65 × 10^−10^, respectively) [78,138].

The identification of genetic variants in immune response genes, including polymorphisms within HLA region and cytokine-related genes such as *IL-1B* and *TNF-α*, may enable the more accurate stratification of women at increased risk of persistent HPV infection [15]. As the majority of infections are transient, an improved understanding of host genetic variations allows the identification of subgroups of women at higher risk of progression to CIN or CxCa [214]. This facilitates the possibility of implementation of intensified surveillance and the differentiation of individuals at higher or lower risk of developing disease [15].

Furthermore, advances in genetic profiling could support the development of personalized screening strategies by incorporating identified genetic variants into polygenic risk scores (PRSs) and then into clinical practice [215]. However, PRSs developed in one ancestry may show reduced predictive performance when applied to another ancestry because differences in linkage disequilibrium structure can alter the relationship between genotyped or tagging variants and the underlying causal variants. This approach may allow for more precise risk stratification and the identification of patients at increased risk of developing CxCa as targets for cervical screening and HPV vaccination, potentially improving both clinical and cost-effectiveness [15,216]. Recent evidence indicates that much of the predictive power of cervical cancer PRSs is driven by variants within the HLA region, underlining the critical role of HLA-mediated immune response to persistent HPV infection in cervical carcinogenesis. These findings also emphasize the importance of using appropriate population-specific linkage disequilibrium reference panels when constructing PRSs, particularly for highly polymorphic regions such as HLA [211]. For instance, a PRS-based study conducted by Tisler et al. showed that women in the highest risk percentile (top 20%) had a 2.32- (BayesRR-RC) to 2.50- (LDpred) fold increased risk of developing CxCa compared to those in the lowest risk group [215]. Importantly, PRS can also highlight individuals in whom the disease may occur substantially earlier. Women within the top 5% of risk reached the same cumulative cancer risk by age 55, approximately 15 years earlier than the population average [215].

Beyond its viral etiology, cervical cancer represents a disease contributed by complex interactions. It mainly develops through a multistep process, primarily starting from HPV infection and progressing through preinvasive lesions to invasive cancer [217]. As mentioned above, host innate and adaptive immune responses play a central role in the course of infection. Research on immune-related genes sheds light on the host–virus interactions, revealing the role of processes such as antigen presentation by HLA molecules and type 2 helper T cells (Th2) cytokine polarization [218,219]. Differences in these mechanisms may explain why some women successfully eliminate the virus, while others experience persistent infection and progression to cancer, and could also guide the development of new therapeutic strategies [15].

### 4.2. Current Limitations and Challenges

Nevertheless, despite recent progress in identifying genetic factors associated with CxCa risk, substantial challenges persist. It is noticeable that for some genes, research results are inconsistent across different populations and study cohorts. For example, the HLA-DQB1*06:02 variant has been associated with an increased risk of developing cervical cancer in some studies, while others have attributed a protective effect [145,146,149]. Similar discrepancies have also been observed for *HLA-DRB1* (*15:01), *IL-10* (rs1800896, rs1800872), *IL-6* (rs1800797), *IL-4R* (rs1805010), *IL-1B* (rs16944), *TLR4* (rs1927911), *TLR9* (rs352140), *CCR2* (rs1799864), *TNF-α* (rs1800629, rs361525), *ICAM-1* (rs5498), *MPO* (rs2333227), *TGFB1* (rs1800469), and *NFKB1* (rs28362491) [32,33,34,38,39,52,53,54,55,61,63,76,77,78,79,81,82,83,92,94,96,97,98,99,100,101,102,103,104,148,174,179,180,184,185,198,199,200,201].

These discrepancies may result from many factors. One of the most important is genetic variation. The same variant may be in linkage disequilibrium with different alleles across populations, which can influence its observed biological effect and association with disease occurrence. Because the main part of the research was conducted in Asian cohorts (e.g., [30,32,52,59,61,62,66]), this restricts the applicability of these findings to other ethnic groups such as European populations. For instance, while association within the HLA region tend to replicate across diverse populations, many non-HLA variants appear to be population-specific and fail to show consistent effects [151]. This challenge is further compounded by small or moderate sample sizes, which reduce statistical power and hinder the detection of low-penetrance variants [15,151]. Hence, the validation of these associations across diverse populations is essential, highlighting the importance of large international collaborations for clinically relevant conclusions [15,220].

Another important limitation concerns the functional interpretation of genetic risk loci. Most associated variants lie within non-coding regulatory regions, making their biological relevance difficult to determine [151]. Rare coding variants in genes such as *MICA* or *HLA-B* may directly affect protein function, but the majority seem to be regulatory variants [221,222]. Progress in understanding the functional impact of risk loci is still limited, as only a few variants have been examined through fine-mapping or validated using advanced approaches such as eQTL analysis [223].

Moreover, many studies lack comprehensive data on environmental and lifestyle factors, such as smoking or obesity, which hinders the full understanding of gene–environment interactions and can also play a crucial role in the variability observed across different analyses and populations [15]. The interaction with the HPV virus also remains a concern. The lack of information regarding the type of virus or its genetic variants in many studies makes it difficult to determine whether a given polymorphism primarily influences susceptibility to HPV, the body’s ability to eliminate viruses, or the risk of progression following disease (e.g., [52,85,94,119]). An additional challenge in standardizing results is the fact that many studies, including GWAS, often focused on precancerous lesions and dysplasia (e.g., CIN) rather than solely on invasive cervical cancer [151]. Although some variants (e.g., HLA) have been shown to influence risk even at the dysplasia stage, combining different disease stages in analyses may also lead to variations in the reported results [118,222,224].

Importantly, the evidence summarized in this review derives from studies with different levels of methodological robustness. A limited number of associations reported for cervical cancer susceptibility have been supported by genome-wide association studies (GWAS), which provide an unbiased approach to variant discovery and generally offer higher statistical power and reproducibility. In contrast, the majority of reported associations, particularly for genes such as *IL10*, *TNF-α*, *TLRs*, and many HLA loci, originate from candidate gene studies with inherently higher susceptibility to false-positive findings, population stratification, and limited reproducibility. Finally, a substantial proportion of reported variants have been identified in single or a small number of independent studies without consistent replication across populations. This stratification of evidence highlights that the strongest support currently exists for loci replicated across multiple independent cohorts, particularly within the HLA region, whereas many non-HLA candidate gene associations require further validation in large, well-powered, multi-ethnic studies, and ideally confirmation through GWAS-level evidence.

Collectively, these limitations may lead to heterogeneity in reported effect sizes and, in some cases, to the over- or underestimation of true genetic associations.

### 4.3. Future Perspectives

Future research should focus on translating the accumulating evidence on immune response gene polymorphisms into a more integrated understanding of cervical cancer susceptibility in the context of HPV infection. As highlighted in this narrative review, variants within key immune pathways including HLA genes, cytokines, Toll-like receptors, and interferon-related genes appear to modulate host responses to HPV, influencing viral clearance, persistence, and progression to cervical cancer. However, their clinical applicability remains limited.

A major direction for future work is the refinement and validation of polygenic risk scores (PRSs) that incorporate immune-related genetic variants to improve individual risk stratification. Current evidence suggests that much of the predictive performance of PRSs is driven by HLA-region variants, underscoring the central role of antigen presentation in HPV-associated carcinogenesis [198]. For PRSs to become clinically meaningful, large-scale validation in multi-ethnic cohorts is essential, particularly given that most available data derive from Asian populations [30,32,52,59,61,62,66]. A major challenge is the limited transferability of PRSs across populations, as differences in population-specific linkage disequilibrium (LD) structures can alter the relationship between risk variants and their correlated markers. This issue may be particularly pronounced within the highly polymorphic HLA region and should therefore be carefully considered when developing and validating PRSs to ensure their reproducibility across populations [198].

Future studies should also aim to integrate genetic susceptibility with viral, environmental, and clinical determinants of disease, including HPV persistence, smoking, and other host-related risk factors, to better reflect the multifactorial nature of cervical carcinogenesis [195,205]. Such integrative approaches may improve the ability to identify women at highest risk of persistent infection and progression to cervical intraepithelial neoplasia or invasive cervical cancer, thereby supporting more personalized prevention and screening strategies [151].

The functional characterization of identified susceptibility loci remains a critical priority. Most associated variants are located in non-coding regulatory regions, and their biological effects on immune regulation and host–HPV interactions are still insufficiently understood [205]. Future work should therefore prioritize fine-mapping, eQTL analyses, and experimental validation to clarify the mechanisms by which these variants influence immune responses, particularly in pathways involved in antigen presentation and inflammatory signaling [207,208,209]. Together, these efforts will be essential for bridging the gap between statistical genetic associations and mechanistic insight into HPV-driven cervical carcinogenesis.

Importantly, while the identified genetic variants and emerging polygenic risk score approaches show promise for improving risk stratification, their current clinical utility remains limited [151]. At present, there is insufficient evidence to support the routine use of individual polymorphisms or PRS models for guiding cervical cancer screening intervals, predicting vaccine response, or determining the clinical management of HPV-positive women [225]. However, in the future, the integration of validated genetic risk markers with HPV status and clinical risk factors may enable the more refined stratification of HPV-positive individuals, including the identification of women at a higher risk of persistent infection and progression to CIN or invasive cervical cancer. Such an approach could ultimately support more personalized screening strategies and prevention programs, although this will require large-scale prospective validation in diverse populations before clinical implementation [151,226].

### 4.4. Conclusions

This narrative review summarizes current evidence on immune response gene polymorphisms and their contribution to cervical cancer susceptibility in the context of persistent HPV infection. The available data indicate that host genetic variation, particularly within HLA genes and other immune-related pathways such as cytokine signaling, Toll-like receptors, and interferon responses, modulates key processes involved in HPV clearance, persistence, and progression to cervical cancer.

Despite substantial progress in identifying susceptibility loci, only a limited number of variants have reached genome-wide significance, and most findings remain derived from relatively small and ethnically homogeneous cohorts. This, together with the population-specific genetic architecture and the incomplete functional annotation of predominantly non-coding variants, continues to limit the clinical translation of current genetic associations.

Overall, cervical cancer emerges as a multifactorial disease driven by complex interactions between viral infection and host immune genetics. While current evidence does not yet support the routine clinical implementation of genetic risk profiling, it provides important insights into the molecular and immunological mechanisms underlying HPV-mediated carcinogenesis. Future large-scale multi-ethnic studies combined with functional validation will be essential to determine the true translational value of immune-related genetic variants and to support the development of more precise, biology-driven prevention strategies.

## Figures and Tables

**Table 1:** Immune response gene polymorphisms supported by multiple independent studies in cervical cancer susceptibility.

Gene	Polymorphism/Allele	Effect	Number of Independent Studies	Study Populations	Biological Relevance	Ref.
*TLR9*	rs187084	↑ risk	2	Indian, Chinese	HPV recognition	[34,37]
*IL1RN*	VNTR	↑ risk	2	Indian, Portuguese	Inflammation regulation	[54,74]
*IL-6*	rs1800795	↑ risk	6	Lithuanian, Iran, Indian, Chinese, Brazilian	Cytokine signaling	[56,57,58,59,60,61]
*IL-6*	rs2069837	↑ risk	2	Chinese	Cytokine signaling	[59,62]
*IL-12B*	rs3212227	↑ risk	3	Polish, Indian, Chinese	Antiviral immunity	[64,65,66]
*IL-17A*	rs2275913	↑ risk	3	Chinese	Chronic inflammation	[69,70,71]
*IFNG*	rs2430561	↑ risk	2	Chinese, Indian	Antiviral response	[110,111]
*HLA-DRB1*	rs9272143	↓ risk	2	Bangladeshi, Swedish	Antigen presentation	[119,128]
*HLA-DRB1*	*15	↑ risk	2	Tunisian, Mexican	Antigen presentation	[131,133]
*HLA-DRB1*	*13:01	↓ risk	2	South African, Swedish	Antigen presentation	[63,129]
*HLA-DPB1*	*13:01	↑ risk	2	Taiwanese, Chinese	Antigen presentation	[139,140]
*HLA-DQB1*	*03:01	↑ risk	4	South African, South Indian, Swedish, US	Antigen presentation	[125,129,132,146]
*ERAP1*	rs27044	↓ risk	2	Chinese, Javanese	Antigen processing	[156,159]
*ERAP1*	rs26653	↓ risk	2	Chinese, Javanese	Antigen processing	[156,159]
*CTLA4*	rs5742909	↑ risk	3	Brazilian, Taiwanese, Polish	T-cell inhibition	[164,165,166]
*CD28*	rs3116496	↑ risk	2	Chinese, Polish	T-cell activation	[166,170]

↑—increase; ↓—decrease.

**Table 2:** Summary of immune response gene polymorphisms associated with HPV-related outcomes.

Gene	SNP/Allele	Population	HPV-Related Endpoint	Association	OR (95% CI)	*p*-Value	Ref.
*TLR4*	rs1927911	North Indian	HPV infection	Increased risk	CT: 1.90 (1.06–3.41) T: 1.65 (1.07–2.55)	0.032 0.023	[34]
*TLR4*	rs4986790	North Indian	HPV infection	Increased risk	AG: 1.96 (1.06–3.64) G: 1.79 (1.06–3.03)	0.033 0.031	[34]
*TLR9*	rs187084	North Indian	HPV infection	Increased risk	TC: 2.92 (1.51–5.64) C: 1.54 (1.03–2.30)	0.001 0.036	[34]
*TLR9*	rs352140	Chinese Han	HPV16-positive CxCa	Increased risk	GA/AA: 13.85 (1.76–108.84)	0.002	[39]
*IL-4R*	rs1805010	North Macedonia	Progression of HPV-positive CIN	Protective effect	AA: 0.39 (0.14–1.11)	0.03	[81]
*IL-6*	rs1800795	Lithuanian	HPV infection	Increased risk	CC: 4.03 (1.56–10.41)	0.002	[56]
*IL-10*	rs1800872	North Macedonia	HPV-positive CIN and CxCa	Increased risk	CC: 2.08 (1.24–3.49)	0.005	[81]
*IL-10*	rs1800896	Japanese	HPV infection	Increased risk	Not reported	0.04	[79]
*IL-17A*	rs2275913	Chinese	HPV16/18-positive CxCa	Increased risk	AA: 3.23 (1.05–13.23)	0.03	[69]
*IL-17A*	rs2275913	Chinese	HPV16/18-positive CxCa	Increased risk	AA: 4.11 (1.14–22.33)	0.02	[70]
*IL17RA*	rs879576	US	HPV16-positive cervical SCC	Increased risk	1.91 (1.30–2.79)	0.018	[87]
*TNF-α*	rs1800629	Indian	HPV16 infection	Increased risk	A: 3.10 (1.60–6.03)	<0.01	[102]
*MICA*	rs2844511	German	HPV infection	Protective effect	0.79 (0.63–0.99)	0.044	[118]
*HLA-B*	*44	Swedish	HPV16 L83V-associated invasive CxCa	Increased risk	3.50 (1.10–11.1)	0.04	[123]
*HLA-B*	*51	Swedish	HPV16 L83V-associated invasive CxCa	Increased risk	4.20 (1.19–14.69)	0.04	[123]
*HLA-B*	*57	Swedish	HPV16 L83V-associated invasive CxCa	Increased risk	4.67 (1.20–18.6)	0.05	[123]
*HLA-G*	rs1063320	Taiwanese	HPV16-positive CSCC	Increased risk	CC: 2.19 (1.35–3.55) C: 1.48 (1.13–1.92)	0.001 0.003	[126]
*HLA-G*	rs12722477	Taiwanese	HPV16-positive CSCC	Increased risk	CC: 2.88 (1.30–6.36) C: 1.69 (1.25–2.28)	0.004 0.0005	[126]
*HLA-DRB1*	rs9357152	German	HPV16-positive CxCa	Increased risk	1.33 (1.03–1.70)	0.03	[127]
*HLA-DRB1*	*07	Chinese Han	HPV16-positive CSCC	Increased risk	2.64 (1.58–6.02)	0.022	[134]
*HLA-DRB1*	*10:01	US	HPV16-positive CSCC	Increased risk	7.30 (1.50–36.70)	<0.05	[132]
*HLA-DRB1*	*11:01	US	HPV16-positive CSCC	Increased risk	2.40 (1.30–4.60)	<0.05	[132]
*HLA-DRB1*	*03:01	US	HPV16-positive CSCC	Protective effect	0.60 (0.40–1.00)	<0.05	[132]
*HLA-DRB1*	*13:01	South African	HPV co-infected CxCa	Protective effect	0.18 (0.02–0.75)	0.012	[129]
*HLA-DPB1*	*05:01	Taiwanese	HPV16-positive CSCC	Protective effect	0.65 (0.52–0.80)	0.0007	[139]
*HLA-DPB1*	*13:01	Taiwanese	HPV16-positive CSCC	Increased risk	1.83 (1.31–2.57)	0.004	[139]
*HLA-DPB1*	*13:01	Chinese	HPV16-positive CxCa	Increased risk	3.34 (1.25–8.90)	0.011	[140]
*HLA-DQA1*	rs9272117	German	HPV infection	Protective effect	0.74 (0.58–0.96)	0.022	[118]
*HLA-DQB1*	*03:01	South African	HPV co-infected CxCa	Increased risk	5.60 (1.90–16.90)	0.001	[129]
*HLA-DQB1*	*06:02	South African	HPV co-infected CxCa	Increased risk	4.50 (2.20–9.00)	<0.0001	[129]
*HLA-DQB1*	*03:01	US	HPV16-positive CSCC	Increased risk	1.50 (1.00–2.20)	<0.05	[132]
*HLA-DQB1*	*03:02:01	Chinese	HPV16 E6 prototype-positive CxCa	Increased risk	10.87 (3.16–37.48)	<0.0001	[142]
*HLA-DQB1*	*06:01:01	Chinese	HPV16 E6 prototype-positive CxCa	Increased risk	5.95 (1.87–18.89)	0.002	[142]
*HLA-DQB1*	*06:01:01	Chinese	HPV16-positive cervical epithelial cell carcinoma	Increased risk	3.74 (2.33–5.99)	<0.0001	[145]
*HLA-DQB1*	rs6457617	Indian	HPV16/18-positive CxCa	Increased risk	2.45 (1.29–4.68)	0.003	[148]
*HLA-DQB1*	*05:02:01	Chinese	HPV16-positive cervical epithelial cell carcinoma	Protective effect	0.22 (0.12–0.41)	<0.0001	[145]
*HLA-DQB1*	*06:02	Uighur	HPV16-positive ICC	Protective effect	0.55 (0.36–0.84)	0.007	[149]
*CTLA4*	rs5742909	Brazilian	HSIL development in HPV-infected women	Increased risk	CT: 2.95 (1.28–6.81)	0.012	[164]
*CTLA4*	rs5742909	Taiwanese	HPV16-positive CSCC	Increased risk	CT: 1.99 (1.16–3.42)	0.03	[165]
*CXCR4*	rs2228014	Brazilian	HPV infection	Increased risk	CT: 2.01 (1.03–3.87)	<0.05	[177]
*CXCL12*	rs1801157	Brazilian	HPV infection	Increased risk	GA: 3.01 (1.77–5.08) AA: 18.03 (4.87–66.71)	<0.01 <0.01	[177]
*OPN*	rs17524488	Chinese	HPV-positive CxCa	Increased risk	GG: 1.80 (1.33–2.22)	<0.001	[182]
*OPN*	rs11730582	Chinese	HPV-positive CxCa	Protective effect	CC: 0.21 (0.19–0.47)	<0.001	[182]
*RAGE*	rs2070600	Chinese	HPV infection	Increased risk	GS: 1.74 (1.15–2.64) SS: 1.68 (1.07–2.64)	0.01 0.02	[189]
*P2RX7*	rs17525809	Taiwanese	HPV-16 positive CSCC	Increased risk	10.2 (1.39–87.8)	0.002	[190]
*FOXP3*	rs2294021	Chinese	HR-HPV risk	Protective effect	TC: 0.59 (0.37–0.93)	0.023	[197]
*FOXP3*	rs3761549	Chinese	HR-HPV risk	Protective effect	GA: 0.46 (0.29–0.74)	0.001	[197]

## Data Availability

All relevant data are included in the manuscript.

## Supplementary Materials

The following supporting information can be downloaded at: https://www.mdpi.com/article/10.3390/ijms27209384/s1.

## Abbreviations

The following abbreviations are used in this manuscript:

CxCa	Cervical cancer
HPV	Human papillomavirus
GWAS	Genome Wide Association Study
TLR	Toll-like receptor
SNP	Single nucleotide polymorphism
VNTR	Variable number of tandem repeats
FIGO	International Federation of Gynecology and Obstetrics
HSIL	High-grade squamous intraepithelial lesion
ICC	Invasive cervical cancer
TNF	Tumor necrosis factor
IFN	Interferon
CSCC	Cervical squamous cell carcinoma
MHC	Major histocompatibility complex
APM	Antigen-processing machinery
CIN	Cervical intraepithelial neoplasia
SIL	Squamous intraepithelial lesions
CAC	Cervical adenocarcinoma
lncRNAs	Long non-coding RNAs
LSIL	Low-grade squamous intraepithelial lesion
eQTL	expression Quantitative Trait Loci
PRS	Polygenic risk score
