# Supplementary material for: Immune Response Gene Polymorphisms and Cervical Cancer Susceptibility
Source: Int J Mol Sci. 2026 Oct 21;27(20):9384. doi: 10.3390/ijms27209384 (PMC13705446; doi:10.3390/ijms27209384)
Supplement: Supplementary file 1 [file ijms-27-09384-s001.zip › ijms-4553986-supplementary.pdf]

Supplementary Tables

Table S1. Summary of toll-like and FAS receptor-encoding genes polymorphisms and their potential contribution to cervical cancer risk

Gene	Polymorphism	Sample size	Unaffected/ affected	Population	Association with HPV	Distribution	OR (95% CI)	*p*-value	Function	Ref
TLR1	rs5833095<br>rs4833095/rs4986790/rs187084	58<br>124	30/28<br>67/57	Brazilian	Not reported<br>Not reported	Genotypes<br>Haplotypes	2.55 (1.08-6.03)<br>CAG: 4.39 (1.13-17.08)	0.034<br>0.035	Involvement in the recognition of bacterial triacylated lipopeptides	(29)
TLR2	-196 to -174 del	300	150/150	North Indian	Not reported	Alleles	1.60 (1.00-2.51)	0.048	Involvement in the recognition of bacterial lipoprotein	(30)
TLR4	rs7873784	1262	842/420	Chinese Han	None<br>None	Alleles<br>Genotypes<br>Genotypes	1.65 (1.23-2.12)<br>CG: 1.49 (1.25-1.87)<br>GG: 2.10 (1.41-2.86)	<0.001<br><0.001<br><0.001	Recognition of lipopolysaccharide molecules	(32)
TLR4	rs1927911<br><br>rs11536889<br>rs4986790	753	423/330	Indian	Associated<br>Associated<br>None<br>Associated	Alleles<br>Genotypes<br>Genotypes<br>Alleles	1.64 (1.11-2.41)<br>2.25 (1.33-3.81)<br>1.95 (1.15-3.31)<br>1.69 (1.04-2.75)	0.013<br>0.003<br>0.013<br>0.033		(34)

					None<br>Associated	Haplotypes<br>Haplotypes	GTAC: 1.77 (1.00-3.13)<br>GCAG: 0.39 (0.19-0.79)	0.04<br>0.0076		
TLR4	rs4986791	300	150/150	North Indian	Not reported	Genotypes	2.51 (1.03-6.12)	0.044		(30)
TLR9	rs187084	753	423/330	Indian	None<br>Associated<br>Associated	Genotypes<br>Alleles<br>Haplotypes	2.04 (1.01-4.13)<br>1.50(1.04-2.14)<br>GATC: 3.95 (1.15-13.50)	0.049<br>0.029<br>0.019	Recognition of unmethylated CpG viral DNA	(34)
TLR9	rs187084	1409	715/694	Chinese	Not reported<br>Not reported	Genotypes<br>Genotypes	TC: 1.28 (1.01-1.62)<br>TC/CC: 1.24 (1.01-1.53)	0.037<br>0.049		(37)
TLR9	rs352140	1262	842/420	Chinese Han	None<br>None	Genotypes<br>Genotypes	GA: 1.33 (1.05-1.71)<br>AA: 2.35 (1.57-3.16)	0.01<br><0.001		(32)
TLR9	rs352140	220	100/120	Chinese Han	Associated<br>None<br>Associated	Genotypes<br>Genotypes<br>Alleles	GA+AA: 7.26 (2.10-25.05)<br>GA: 6.93 (1.53-33.30)<br>7.00 (2.42-20.23)	<0.001<br>0.004<br><0.001		(39)
TLR9	rs352140	886	460/426	Polish	Not reported	Genotypes	CT+TT: 1.41 (1.03-1.93)	0.0326		(38)

					Not reported	Genotypes	TT: 1.49 (1.01-2.18)	0.0441		
FAS	rs1800682	394	209/185	Malaysian	Not reported<br><br>Not reported<br><br>Not reported	Genotypes<br><br>Genotypes<br><br>Alleles	AG: 2.75 (1.44-5.23)<br>GG: 1.99 (1.19-3.56)<br>1.76 (1.26-2.46)	<0.002<br><br>0.019<br><br>0.001	Activation of apoptotic signalling	(41)
FAS	rs1800682	352	176/176	Chinese	None<br><br>None<br><br>None	Alleles<br><br>Genotypes<br><br>Genotypes	OR: 1.5 (1.1-2.0)<br>AA: 2.20 (1.20-4.20)<br>AG: 1.30 (0.70-2.20)	0.006<br><br>0.017<br><br>0.017		(42)
FASL	rs763110	942	615/314	Chinese	Not reported	Genotypes	3.05 (1.43-6.52)	0.004	Mediation of activation-induced cell death	(44)

Table S2. Summary of interleukin-related genes polymorphisms and their potential contribution to cervical cancer risk

Gene	Polymorphism	Sample size	Unaffected/ affected	Population	Association with HPV	Distribution	OR (95% CI)	*p*-value	Function	Ref
IL-1A	rs3783553	561	326/235	Chinese	Not reported	Genotypes	0.48 (0.25-0.95)	0.03	Initiation and amplification of inflammatory responses	(88)
IL-1A	rs3783553	743	424/319	Chinese Han	Not reported<br>Not reported<br>Not reported	Alleles<br>Genotypes<br>Genotypes	0.71 (0.57-0.88)<br>II: 0.46 (0.28-0.77)<br>DI/II: 0.68 (0.50-0.91)	0.014<br>0.006<br>0.009		(89)
IL-1B	rs16944	480	228/252	Bangladeshi	Not reported<br>Not reported<br>Not reported	Genotypes<br>Genotypes<br>Alleles	GG: 1.56 (0.99-2.45)<br>AG+GG: 1.71 (1.11-2.63)<br>1.30 (1.01-1.6.8)	0.006<br>0.015<br>0.046	Involvement in acute and chronic inflammation	(53)
IL-1B	rs16944	312	162/150	Indian	Not reported<br>Not reported	Genotypes<br>Alleles	2.77 (1.21-6.41)<br>1.83 (1.28-2.61)	0.012<br>0.0007		(54)
IL-1B	rs16944	546	364/182	Korean	Not reported	Genotypes	CT: 2.83 (1.52-5.28)	<0.001<br><0.005		(55)

					Not reported	Genotypes	CT+TT: 2.42 (1.31-4.46)			
IL-1B	rs16944	569	302/267	Chinese Uygur	Not reported	Genotypes	GG: 1.89 (1.15-3.11)	0.039		(52)
	rs1143627				Not reported	Genotypes	AA: 1.88 (1.14-3.10)	0.042		
	rs3136558				Not reported	Genotypes	GA: 0.61 (0.42-0.89)	0.033		
	rs114360				Not reported	Genotypes	AG: 0.68 (0.46-0.99)	0.042		
	rs1143630/rs1143627/rs16944				Not reported	Haplotypes	TGA: 0.56 (0.40-0.81)	0.002		
IL1RN	VNTR	312	162/150	Indian	Not reported	Genotypes	2/2: 2.89 (1.15-7.48)	0.02	Modulation of host immune response	(54)
					Not reported	Genotypes	1/2: 3.3 (1.94-5.74)	4.9×10^{-6}		
IL1RN	VNTR	536	196/340	Portuguese	Not reported	Genotypes	1.85 (1.07-3.19)	0.025		(74)
IL1R2	rs719250	533	286/247	Chinese Uygur	Not reported	Alleles	1.44 (1.08-1.91)	0.013	Inhibition of inflammation	(85)
					Not reported	Genotypes	2.00 (1.39-2.87)	6×10^{-4}		
	rs3218896				Not reported	Alleles	1.55 (1.08-2.23)	0.017		
					Not reported	Genotypes	1.32 (0.26-6.60)	0.034		
	rs11674595/rs4851527				Not reported	Haplotypes	TC: 0.70 (0.52-0.93)	0.015		

	rs719250/rs3218896				Not reported	Haplotypes	TC: 1.64 (1.12-2.39)	0.010		
IL-4R	rs1801275	480	228/252	Bangladeshi	Not reported<br>Not reported<br>Not reported	Alleles<br>Genotypes<br>Genotypes	1.59 (1.21-2.10)<br>GG: 2.45 (1.40-4.27)<br>GA+GG: 1.49 (1.04-2.14)	<0.0009<br>0.002<br>0.030	Receptor involved in Th2 responses and IgE class switching	(53)
IL-4R	rs1805010	247	113/134	North Macedonia	Associated	Genotypes	0.39 (0.14-1.11)	0.03		(81)
IL-4R	rs1805010	1594	288/1306	Swedish	Not reported	Genotypes	1.91 (1.27-2.86)	0.0072		(92)
IL-6	rs1800795	173	84/89	Lithuanian	Associated<br>Associated	Genotypes<br>Alleles	5.37 (2.08-13.84)<br>2.26 (1.19-4.29)	<0.001<br>0.012	Inflammation and B-cell differentiation	(56)
IL-6	rs1800795	264	140/124	Iran	Not reported<br>Not reported	Genotypes<br>Alleles	3.23 (1.13-9.24)<br>1.92 (1.27-2.90)	0.029<br>0.002		(57)
IL-6	rs1800795	360	200/160	Indian	Not reported	Genotypes	3.16 (1.26-7.92)	0.014		(58)
IL-6	rs1800795<br>rs2069837<br>rs1800795/rs2069837	1088	728/360	Chinese	Not reported<br>Not reported<br>Not reported	Genotypes<br>Genotypes<br>Haplotypes	1.60 (1.24-2.19)<br>1.49 (1.19-2.07)<br>3.35 (2.01-4.78)	<0.05<br><0.05<br><0.001		(59)

IL-6	rs2069837	3340	1756/1584	Chinese	Not reported	Genotypes	AG: 1.29 (1.09-1.53)	0.003		(62)
IL-6	rs1800795	309	253/56	Brazilian	Not reported	Genotypes	GC+CC: 1.88 (1.10-3.40)	0.035		(60)
IL-6	rs1800795<br><br>rs1800797	1036	518/518	Chinese	Not reported<br>Not reported<br>Not reported	Genotypes<br>Alleles<br>Genotypes	1.52 (1.06-2.19)<br>1.21 (1.02-1.44)<br>1.91 (1.16-3.13)	0.02<br>0.03<br>0.01		(61)
IL-6	rs1800797	480	228/252	Bangladeshi	Not reported<br>Not reported<br>Not reported<br>Not reported	Genotypes<br>Genotypes<br>Genotypes<br>Alleles	AA: 9.86 (2.76-35.21)<br>AG: 8.13 (5.27-12.55)<br>AG+AA: 8.25 (5.40-12.60)<br>4.99 (3.49-7.13)	0.0004<br><0.0001<br><0.0001<br><0.0001		(53)
IL-6	rs1800797	2669	1715/954	Swedish	Not reported<br>Not reported	Genotypes<br>Alleles	0.77 (0.61-0.98)<br>0.88 (0.79-0.99)	0.03<br>0.03		(63)
IL-10	rs1800896<br>rs1800896/rs1800871	309	167/142	Brazilian	Not reported<br>Not reported	Genotypes<br>Diplotypes	0.35 (0.14-0.88)<br>0.29 (0.12-0.70)	0.0266<br>0.0045	Involvement in anti-inflammatory responses	(76)
IL-10	rs1800896	392	200/192	Indian	Not reported	Genotypes	AG: 2.35 (1.54-3.58)	0.0006		(77)

					Not reported<br>Not reported	Genotypes<br>Alleles	AG+GG: 2.03 (1.36-3.04)<br>1.39 (1.02-1.88)	0.0005<br>0.036		
IL-10	rs1800872<br>rs1800896<br>rs1800872/rs1800896<br>rs1800872/rs1800896	444	204/240	Bangladeshi	Not reported<br>Not reported<br>Not reported<br>Not reported<br>Not reported	Genotypes<br>Alleles<br>Genotypes<br>Haplotypes<br>Haplotypes	AA: 2.75 (1.53-4.93)<br>1.55 (1.19-2.03)<br>GG: 3.48 (1.46-8.31)<br>AA: 1.56 (1.19-2.03)<br>CA: 0.42 (0.31-0.57)	0.0007<br>0.001<br>0.005<br>0.001<br>2.42×10^{-8}		(78)
IL-10	rs1800872	247	113/134	North Macedonia	Associated<br>Associated	Genotypes<br>Alleles	CC vs CA+AA: 2.08 (1.24-3.49)<br>1.73 (1.10-2.60)	0.005<br>0.01		(81)
IL-10	rs1800871	330	165/165	Chinese	Not reported<br>Not reported	Genotypes<br>Genotypes	TT: 2.23 (1.16-4.30)<br>CT+TT: 1.69 (1.04-2.76)	0.01<br>0.03		(84)
IL-10	rs1800896	277	173/104	Japanese	Associated	Genotypes	3.90 (2.10-7.30)	<0.05		(79)
IL-10	rs1800872	1590	606/984	Dutch	Not reported<br>Not reported	Genotypes<br>Genotypes	CA: 1.38 (1.11-1.72) 	0.004<br>0.004		(83)

							CA+AA: 1.37 (1.10-1.69)			
IL-12A	rs568408	808	404/404	Chinese	Not reported	Genotypes	1.43 (1.06-1.93)	<0.05	Involvement in Th1 differentiation and promotion of cell-mediated immunity	(66)
IL-12B	rs3212227	855	450/405	Polish	Not reported<br><br>Not reported<br><br>Not reported	Genotypes<br><br>Genotypes<br><br>Genotypes	AC+CC: 1.64 (1.24-2.15)<br>CC: 2.59 (1.18-5.69)<br>AC: 1.57 (1.19-2.08)	0.0004<br><br>0.0143<br><br>0.0016	Involvement in Th1 responses and IFN-γ production	(64)
IL-12B	rs3212227	400	200/200	Indian	Not reported<br><br>Not reported	Genotypes<br><br>Genotypes	AC: 1.80 (1.17-2.78)<br>AC+CC: 1.71 (1.11-2.63)	0.006<br><br>0.01		(65)
IL-12B	rs3212227	808	404/404	Chinese	Not reported	Genotypes	1.30 (0.97-1.75)	<0.05		(66)
IL-12B	IVS2 -912G/A SNP<br>IVS2 -912G:IVS4 +314A	345	191/154	Korean	Not reported<br><br>Not reported	Genotypes<br><br>Haplotypes	8.77 (1.11-6.69)<br>GA: 2.71 (1.54-4.77)	0.040<br><br><0.001		(67)
IL-16	rs1131445	672	334/338	Chinese	Not reported	Genotypes	TC+ CC: 1.51 (1.18-1.93)	0.001	Involvement in CD^{4+} T-cell	(86)

						Genotypes<br><br>Genotypes	CC: 1.88 (1.31-2.73)<br>TC: 1.42 (1.09-1.85)	0.0006<br><br>0.008	chemoattraction	
IL-17A	rs3748067	312	156/156	Bangladeshi	Not reported	Genotypes	1.79 (1.09-2.92)	0.021	Pro-inflammatory responses and neutrophil recruitment	(68)
IL-17A	rs2275913	704	352/352	Chinese	Associated	Genotypes	1.64 (1.20-2.24)	0.001		(69)
IL-17A	rs2275913	528	264/264	Chinese	Associated	Genotypes	2.34 (1.24-4.49)	0.005		(70)
IL-17A	rs2275913	774	463/311	Chinese	Not reported<br><br>Not reported	Genotypes<br><br>Alleles	1.72 (1.15-2.57)<br>1.32 (1.07-1.62)	0.008<br><br>0.008		(71)
IL17RA	rs879576	2367	1109/1258	US	Associated	Alleles	1.91 (1.30-2.79)	0.018	IL-17-mediated inflammatory signalling	(87)
IL-18	rs187238	400	200/200	North Indian	Not reported<br><br>Not reported	Genotypes<br><br>Genotypes	GC: 1.80 (1.17-2.76)<br>GC+CC: 1.60 (1.09-2.50)	0.006<br><br>0.01	IFN-γ production and enhancement of Th1 immune response	(88)

IL-27	rs17855750	760	380/380	Chinese Han	Not reported<br><br>Not reported<br><br>Not reported	Genotypes<br><br>Genotypes<br><br>Genotypes	GG: 0.95 (0.72-0.96)<br>TG: 0.77 (0.60-0.86)<br>TG+GG: 0.87 (0.65-0.94)	0.018<br><br>0.031<br><br>0.021	Regulation of Th1 differentiation and modulation of immune responses	(89)

Table S3. Summary of *TNF-α, IFNG* and *IFNA17* polymorphisms and their associations with cervical cancer risk

Gene	Polymorphism	Sample size	Unaffected/ affected	Population	Association with HPV	Distribution	OR (95% CI)	*p*-value	Function	Ref
TNF-α	rs1800629	159	126/133	Bangladeshi	Not reported	Genotypes	AA: 4.55 (1.24-16.60)	0.022	Involvement in inflammation, immune cell activation and apoptosis	(96)
					Not reported	Genotypes	AG: 2.85 (1.20-6.74)	0.017		
					Not reported	Genotypes	AG+AA: 3.31 (1.59-6.91)	0.001		
					Not reported	Alleles	3.37 (1.80-6.32)	0.0002		
	rs1799724				Not reported	Genotypes	TT: 3.26 (1.15-9.28)	0.027		
					Not reported	Genotypes	CT+TT: 2.42 (1.20-4.91)	0.014		
					Not reported	Alleles	2.58 (1.45-4.60)	0.013		
TNF-α	rs1800629	2153	1173/980	Chinese Han	None	Alleles	0.71 (0.56-0.92)	0.009		(94)
	rs361525				None	Alleles	1.69 (1.21-2.38)	0.002		
TNF-α	rs1800629	445	292/153	Iran	Not reported	Genotypes	3.79 (2.4-5.7)	<0.001		(97)
TNF-α	rs361525	2144	1100/1044	Chinese	None	Genotypes	0.39 (0.26-0.59)	<0.01		(82)
					None	Alleles	0.72 (0.55-0.94)	0.015		

	rs1800629				None<br>None	Genotypes<br>Alleles	1.895 (1.344-2.671)<br>1.35 (1.07-1.71)	<0.01<br>0.013		
TNF-α	rs361525<br>rs1800629<br>rs1799964	946	494/452	Chinese	Not reported<br>Not reported<br>Not reported	Alleles<br>Alleles<br>Alleles	3.04 (1.76-5.25)<br>2.58 (1.87-3.56)<br>1.43 (1.07-1.91)	<0.01<br><0.01<br><0.05		(98)
TNF-α	rs1799724	699	351/348	Chinese	Associated	Alleles	3.99 (2.90-5.51)	<0.05		(106)
TNF-α	rs1800629	761	399/362	Polish	None	Genotypes	1.60 (1.02-2.51)	0.042		(99)
TNF-α	rs1800629	428	223/205	Portuguese	Not reported	Genotypes	1.87 (1.20-2.91)	0.005		(100)
TNF-α	rs1800629	312	162/150	Indian	Not reported<br>Not reported	Genotypes<br>Alleles	AG+AA: 2.24 (1.14-4.40)<br>2.05 (1.12-3.77)	0.018<br>0.012		(101)
TNF-α	rs1800629	185	165/120	Indian	Associated	Genotypes	2.80 (1.39-5.49)	<0.01		(102)
TNF-α	rs1800629	439	244/195	Portuguese	Not reported<br>Not reported	Alleles<br>Genotypes	1.88 (1.20-2.94)<br>AG: 1.81 (1.10-2.97)	0.006<br>0.0124		(103)
TNF-α	rs1800629	169	113/56	Posadas	Not reported	Genotypes	2.40 (1.10-5.40)	<0.05		(104)

IFNG	rs2430561	386	200/186	Chinese	Not reported<br>Not reported<br>Not reported	Genotypes<br>Genotypes<br>Genotypes	AA: 2.22 (1.19-4.15)<br>TA: 1.58 (0.86-2.91)<br>1.47 (1.10-1.97)	0.012<br>0.012<br>0.009	Activation of macrophage and promotion of Th1 immune responses	(110)
IFNG	rs2430561	430	230/200	Indian	Not reported<br>Not reported	Genotypes<br>Alleles	2.43 (1.34-4.42)<br>1.54 (1.17-2.03)	0.003<br>0.002		(111)
IFNGA	CA repeat polymorphism	306	153/153	Taiwanese	Not reported	Genotypes	13/18: 0.60 (0.40-1.00)	0.037		(113)
IFNA17	rs9298814	371	198/173	South Korean	Not reported	Genotypes	2.96 (1.53-5.73)	0.001	Mediation of neutrophil recruitment	(114)

Table S4. Summary of MHC class I and II genes' polymorphisms/alleles and their potential contribution to cervical cancer risk

Gene	Polymorphism/ allele	Sample size	Unaffected/ affected	Population	Association with HPV	Distribution	OR (95% CI)	*p*-value	Function	Ref
MICA	rs2844511	2961	1556/1405	German	Associated	Alleles	1.17 (1.04-1.31)	0.008	Activation of immune cells via NKG2D binding	(118)
MICA	rs2516448	2626	1683/943	Swedish	Not reported	Alleles	1.33 (1.19-1.49)	5.8×10^{-7}		(119)
HLA-A	*01:01<br>*30:01<br>*33:03	465	213/252	Chinese	None<br>None<br>None	Alleles<br>Alleles<br>Alleles	2.18 (1.33-3.57)<br>0.53 (0.28-0.99)<br>0.54 (0.30-0.97)	0.002<br>0.046<br>0.037	Antigen presentation to T cells	(120)
HLA-A	*02:06	238	119/119	Japanese	Not reported	Alleles	0.31 (0.15-0.65)	0.002		(121)
HLA-B	rs1056429	2315	1194/1121	German	None	Alleles	0.82 (0.70-0.97)	0.023	Antigen presentation to T cells	(122)
HLA-B	*44<br>*51<br>*57	327	300/27	Swedish	Associated<br>Associated<br>Associated	Alleles<br>Alleles<br>Alleles	3.50 (1.10-11.1)<br>4.20 (1.19-14.69)<br>4.67 (1.20-18.6)	0.04<br>0.04<br>0.05		(123)

HLA-B	*51:01:02<br><br>*51:01:01	354	254/100	Chinese	Not reported<br><br>Not reported	Alleles<br><br>Alleles	26.68 (3.99-209.87)<br>0.12 (0.03-0.52)	3.17×10^{-5}<br>7.01×10^{-4}		(124)
HLA-A-HLA-B	*HLA-A**11-*HLA-B**07<br><br>*HLA-A**03-*HLA-B**35<br><br>*HLA-A**11-*HLA-B**35	95	47/48	South Indian	Not reported<br><br>Not reported<br><br>Not reported	Haplotypes<br><br>Haplotypes<br><br>Haplotypes	3.95 (1.46-11.96)<br><br>0.25 (0.03-1.18)<br><br>0.06 (0.00-0.32)	<0.005<br><br>0.088<br><br>1.47×10^{-4}		(125)
HLA-G	rs1063320<br><br><br>14-bp del/ins<br><br>rs12722477-14-bp del	717	400/317	Taiwanese	Associated<br><br>Associated<br><br>None<br><br>Associated	Genotypes<br><br>Alleles<br><br>Genotypes<br><br>Haplotypes	1.78 (1.18-2.69)<br>1.31 (1.06-1.62)<br>0.70 (0.51-0.95)<br>1.41 (1.09-1.82)	0.004<br><br>0.014<br><br>0.03<br><br>0.009	Immune tolerance	(126)
HLA-DRB1	rs9357152	758	561/197	German	Associated	Alleles	1.33 (1.03-1.70)	0.03	Antigen presentation to $CD4^{+}$ T cells	(127)
HLA-DRB1	rs9272143	288	118/170	Bangladeshi	Not reported	Genotypes	0.46 (0.31-0.70)	0.0004		(128)
HLA-DRB1	rs9272143	2686	1725/961	Swedish	Not reported	Alleles	0.73 (0.65-0.82)	1.6×10^{-7}		(119)

HLA-DRB1	*07	250	94/156	Chinese Han	Associated	Alleles	2.31 (1.08-4.95)	0.041		(134)
HLA-DRB1	*04	95	47/48	South Indian	Not reported	Alleles	2.57 (0.98-7.23)	0.055		(125)
HLA-DRB1	*04:01	2563	1642/921	Swedish	Not reported	Alleles	1.64 (0.86-3.12)	0.01		(63)
HLA-DRB1	*15	276	148/128	Tunisian	Not reported	Alleles	2.69 (1.10-6.72)	0.016		(131)
HLA-DRB1	*15	208	104/104	Mexican	None	Alleles	3.90 (1.60-10.20)	0.002		(133)
HLA-DRB1	*10:01<br>*11:01	696	381/315	US	Associated<br>Associated	Alleles<br>Alleles	5.60 (1.20-26.10)<br>2.40 (1.40-4.20)	<0.05<br><0.05		(132)
HLA-DRB1	*13:01	456	200/256	South African	Associated	Alleles	0.18 (0.02-0.75)	0.012		(129)
HLA-DRB1	*13:01	2563	1642/921	Swedish	Not reported	Alleles	0.43 (0.18-1.00)	<0.001		(63)
HLA-DRB1	*13:02:01<br>*04:06	1383	926/457	Korean	Not reported<br>Not reported	Alleles<br>Alleles	0.48 (0.27-0.84)<br>0.44 (0.20-0.97)	0.001<br>0.033		(136)
HLA-DRB1	*10	95	47/48	South Indian	Not reported	Alleles	0.22 (0.07-0.62)	0.003		(125)
HLA-DRB1	*15:01	599	312/287	Chinese Han	None	Alleles	0.31 (0.12-0.81)	0.024		(149)
HLA-DRB1	*03:01	696	381/315	US	Associated	Alleles	0.70 (0.50-0.90)	<0.05		(132)

HLA-DRB1	*15:01	2563	1642/921	Swedish	Not reported	Alleles	1.49 (0.94-2.35)	0.0008		(63)
HLA-DPA1	rs3077	4426	2109/2317	Chinese	Not reported	Genotypes	1.51 (1.32-1.71)	5.65 x 10^{-10}		(138)
HLA-DPB1	*05:01<br><br>rs927753<br><br>*13:01	1149	676/473	Taiwanese	Associated<br><br>None<br><br>Associated	Alleles<br><br>Alleles<br><br>Alleles	0.72 (0.61-0.86)<br>1.25 (1.05-1.49)<br>1.83 (1.31-2.57)	0.001<br><br>0.03<br><br>0.004	Antigen presentation to $CD4^+$ T cells	(139)
HLA-DPB1	rs927753	4426	2109/2317	Chinese	Not reported	Genotypes	1.29 (1.12-1.49)	3.57 x 10^{-4}		(138)
HLA-DPB1	*13:01	231	98/133	Chinese	Associated	Alleles	3.79 (1.53-9.42)	0.002		(140)
HLA-DPB1	*02:02	214	88/126	Chinese	Not reported	Alleles	3.65 (1.04-12.80)	0.031		(141)
HLA-DPB1	*04:02	5018	3984/1034	Swedish	Not reported	Alleles	0.75 (0.63-0.89)	7.0 x 10^{-4}		(143)
HLA-DPB2	rs3117027	5018	3984/1034	Swedish	Not reported	Alleles	1.29 (1.16-1.43)	1.9 x 10^{-6}	Pseudogene	(143)
HLA-DQA1	rs9272117	4037	1556/2481	German	Associated	Alleles	0.89 (0.79-0.99)	0.036	Presentation of peptides derived from extracellular proteins	(118)
HLA-DQB1	*03:01<br><br>*06:02	456	200/256	South African	Associated	Alleles	4.66 (1.85-11.72)	0.002<br><br>0.002	Presentation of peptides derived from	(129)

					Associated	Alleles	2.68 (1.45-4.96)		extracellular proteins	
HLA-DQB1	*03:01	95	47/48	South Indian	Not reported	Alleles	4.91 (1.48-21.22)	0.007		(125)
HLA-DQB1	*03:01<br><br>*06:02	1594	288/1306	Swedish	Not reported<br><br>Not reported	Alleles<br><br>Alleles	1.45 (1.07-1.95)<br>1.46 (1.09-1.94)	0.02<br><br>0.01		(146)
HLA-DQB1	*03:01	696	381/315	US	Associated	Alleles	1.60 (1.20-2.20)	<0.05		(132)
HLA-DQB1	*03:02:01<br><br>*06:01:01	247	168/79	Chinese	Associated<br><br>Associated	Alleles<br><br>Alleles	10.87 (3.15-37.48)<br>5.95 (1.87-18.89)	<0,0001<br><br>0.002		(142)
HLA-DQB1	*03:03:02	214	88/126	Chinese	Not reported	Alleles	1.85 (1.04-3.27)	0.034		(141)
HLA-DQB1	*06:01:01	542	284/258	Chinese	Associated	Alleles	2.66 (1.86-3.81)	<0.0001		(145)
HLA-DQB1	*02:01	248	173/75	Indian	Not reported	Alleles	4.47 (1.81-11.25)	0.001		(130)
HLA-DQB1	*04:02	115	79/36	Venezuelan	None	Alleles	RR: 5.07 (1.82-14.12)	0.004		(147)
HLA-DQB1	rs6457617	218	111/107	Indian	Associated	Genotypes	2.56 (1.42-4.62)	0.001		(148)
HLA-DQB1	*03:19	456	200/256	South African	None	Alleles	0.11 (0.003-0.74)	0.011		(129)

HLA-DQB1	*06:04:01	1383	926/457	Korean	Not reported	Alleles	0.46 (0.22-0.94)	0.021		(136)
HLA-DQB1	*06	247	173/75	Indian	Not reported	Alleles	0.54 (0.35-0.85)	0.004		(130)
HLA-DQB1	*06:03<br><br>*05:01	1594	288/1306	Swedish	Not reported<br><br>Not reported	Alleles	0.51 (0.33-0.80)<br>0.72 (0.52-0.99)	0.001<br><br>0.04		(146)
HLA-DQB1	*05:02:01	542	284/258	Chinese	Associated	Alleles	0.38 (0.25-0.59)	<0.0001		(145)
HLA-DQB1	*06:02	599	312/287	Chinese	Associated	Alleles	0.48 (0.33-0.72)	<0.05		(149)

Table S5. Summary of APM genes polymorphisms and their association with cervical cancer risk

Gene	Polymorphism	Sample size	Unaffected /affected	Population	Association with HPV	Distribution	OR (95% CI)	*p*-value	Function	Ref
TAP1	rs41549617<br>rs1135216<br>*02:01:01<br>*03:01	2289	1255/1034	Chinese Han	Not reported<br>Not reported<br>Not reported<br>Not reported	Alleles<br>Alleles<br>Alleles<br>Alleles	0.48 (0.29-0.79)<br>0.75 (0.63-0.88)<br>0.75 (0.63-0.89)<br>1.57 (1.18-2.09)	0.003<br>5.0×10^{-4}<br>0.001<br>0.002	Peptide transport	(155)
TAP2	rs241441	2289	1255/1034	Chinese Han	Not reported	Alleles	1.23 (1.09-1.39)	7.0×10^{-4}	Peptide transport	(155)
TAP2	rs4148876	342	136/206	Balinese	Not reported	Alleles	3.84 (2.38-6.19)	<0.001		(156)
TAPBP	rs1059288	1998	1077/921	Chinese	Not reported<br>Not reported	Genotypes<br>Genotypes	GG: 1.48 (1.13-1.92)<br>AG: 1.24 (0.97-1.58)	4.0×10^{-3}<br>9.0×10^{-2}	Peptide loading	(157)
PSMB8	rs2071543	2128	1120/1008	Chinese Han	Not reported<br>Not reported	Genotypes<br>Genotypes	GT: 1.33 (1.09-1.62)<br>TT: 1.10 (0.63-1.93)	0.020<br>0.020	Generation of peptides	(158)
PSMB9	rs17587	2128	1120/1008	Chinese Han	Not reported<br>Not reported	Genotypes<br>Genotypes	GA: 1.34 (1.11-1.62)<br>AA: 1.48 (0.96-2.29)	0.004<br>0.004	Generation of peptides	(158)

ERAP1	rs26618	2334	1262/1072	Chinese	Not reported	Genotypes	CC: 1.53 (1.14-2.05)	0.004	Peptide trimming	(159)
					Not reported	Genotypes	CT: 1.04 (0.88-1.24)	0.016		
	rs27044				Not reported	Alleles	0.84 (0.75-0.94)	0.003		
	rs26653				Not reported	Alleles	0.83 (0.74-0.93)	0.001		
	rs27044/rs30187/rs26618/rs26653/rs3734016				Not reported	Haplotypes	CTTGC: 0.80 (0.71-0.91)	0.001		
ERAP1	rs26653	406	210/196	Javanese	Not reported	Alleles	0.66 (0.44-0.99)	0.042		(156)
	rs30187				Not reported	Alleles	0.64 (0.43-0.96)	0.032		
	rs27044				Not reported	Alleles	0.64 (0.43 – 0.96)	0.031		
	rs10050860/rs27044				Not reported	Haplotypes	TC: 4.35 (1.18-15.86)	0.026		
ERAP2	rs2287988	2334	1262/1072	Chinese	Not reported	Alleles	1.19 (1.06-1.33)	0.004	Peptide trimming	(159)
	rs27044/rs30187/rs26618/rs26653/rs3734016				Not reported	Haplotypes	TTGAT: 0.84 (0.74-0.94)	0.003		
					Not reported	Haplotypes	GAAGT: 1.59 (1.12-2.26)	0.009		

Table S6. Summary of co-stimulatory and immune checkpoint genes polymorphisms and their potential contribution to cervical cancer risk

Gene	Polymorphism	Sample size	Unaffected/ affected	Population	Association with HPV	Distribution	OR (95% CI)	*p*-value	Function	Ref
CTLA4	rs5742909<br><br>rs5742909/ rs231775/ rs3087243	445	340/105	Brazilian	Associated<br><br>Associated	Genotypes<br><br>Haplotypes	4.21 (1.85-9.60)<br>TAG: 3.21 (1.34-7.69)	0.001<br><br>0.009	Inhibition of T cell activity	(164)
CTLA4	rs5742909	522	378/144	Taiwanese	Associated	Genotypes	1.99 (1.16-3.42)	0.03		(165)
CTLA4	rs5742909<br><br><br><br><br><br>4g.*642AT (8_33)	372	225/147	Polish	Not reported<br><br>Not reported<br><br>Not reported<br><br>Not reported<br><br><br>Not reported<br><br><br>Not reported	Genotypes<br><br>Genotypes<br><br>Alleles<br><br>Genotypes<br><br><br>Genotypes<br><br><br>Alleles	TT+CT: 4.44 (2.07-9.53)<br>CT: 3.81 (1.75-8.31)<br>3.84 (2.04-7.26)<br>$(AT)_8/(AT)_8$: 1.93 (1.23-3.03)<br>$(AT)_8/(AT)_{>8}$: 0.58 (0.37-0.92)<br>1.41 (1.04-1.90)	0.00001<br><br>0.0004<br><br>0.00001<br><br>0.004<br><br><br>0.02<br><br><br>0.03		(166)
CTLA4	rs231775	1405	709/696	Chinese Han	Not reported	Genotypes	1.66 (1.13-2.44)	0.008		(167)

PD1	rs2227981	306	250/256	Chinese	Not reported	Genotypes	2.18 (1.37-6.11)	0.009	Inhibition of T cell activity	(168)
CD28	rs3116496	1604	985/619	Chinese	Not reported<br>Not reported	Genotypes<br>Genotypes	TC+CC: 1.66 (1.27-2.17)<br>TC: 1.69 (1.28-2.23)	<0.001<br><0.001	Promotion of T cell activity	(170)
CD28	rs3116496	372	225/147	Polish	Not reported<br>Not reported<br>Not reported	Genotypes<br>Genotypes<br>Genotypes	CC+TC: 2.05 (0.99-4.28)<br>TT: 0.49 (0.23-1.02)<br>1.89 (1.00-3.56)	0.05<br>0.05<br>0.05		(166)
CD83	rs750749	947	307/640	US	None	Genotypes	0.50 (0.19-1.20)	0.02	Immune checkpoint	(171)
CD226	rs727088	729	380/349	Chinese	Not reported	Alleles	1.42 (1.13-1.79)	0.003	Activation of NK and T cells	(172)

Table S7. Summary of other immune-related genes polymorphisms and their association with risk of cervical malignancies

Gene	Polymorphism	Sample size	Unaffected/ affected	Population	Association with HPV	Distribution	OR (95% CI)	*p*-value	Function	Ref
CCR2	rs1799864	1878	1432/446	African	None<br>None	Alleles<br>Genotypes	6.14 (4.79-7.86)<br>3.99 (1.68-9.50)	0.001<br>0.002	Chemokine-mediated cell migration	(175)
CCR2	rs1799864	1594	288/1306	Swedish	Not reported	Genotypes	0.31 (0.12-0.77)	<0.05		(92)
CCR5	rs333	312	162/150	Indian	Not reported	Genotypes	4.43 (1.10-20.6)	0.021	Chemokine-mediated cell migration	(176)
CXCR4	rs2228014	279	187/92	Brazilian	Associated	Genotypes	4.54 (1.96-11.11)	<0.01	Chemokine-mediated cell migration	(177)
CXCL12	rs1801157	279	187/92	Brazilian	Associated	Genotypes	5.96 (1.89-18.72)	<0.05	Regulation of cell trafficking	(177)
CXCL12	rs266085	699	351/348	Chinese Han	None<br>None<br>None	Genotypes<br>Genotypes<br>Alleles	TC: 1.70 (1.15-2.53)<br>TT: 1.78 (1.14-2.77)<br>1.41 (1.14-1.74)	0.008<br>0.010<br>0.001		(106)
ICAM-1	rs5498<br>rs5498/rs3093030/rs281432	998	510/488	Chinese Han	Not reported<br>Not reported	Alleles<br>Haplotypes	0.50 (0.22-0.82)<br>CTG: 0.96 (0.69-1.33)	0.009<br>0.007	Leukocyte adhesion and migration	(179)

ICAM-1	rs5498<br><br>rs281432	381	290/91	Taiwanese	Not reported<br><br>Not reported	Genotypes<br><br>Genotypes	1.39 (0.39-4.90)<br>2.01 (1.34-3.02)	<0.05<br><br>0.04		(180)
OPN	rs17524488<br><br><br><br>rs11730582	1074	774/300	Chinese	Associated<br><br>Associated<br><br>Associated<br><br>Associated	Genotypes<br><br>Genotypes<br><br>Genotypes<br><br>Genotypes	GG: 1.76 (1.14-2.15)<br>GG/GG: 2.14 (1.43-3.02)<br>TT: 0.22 (0.15-0.32)<br>TC: 0.33 (0.22-0.54)	<0.001<br><br><br><br><0.001	Osteoclast regulation and cell adhesion	(182)
MPO	rs2333227	969	493/476	Polish	Not reported	Genotypes	0.72 (0.53-0.97)	0.03	Involvement in neutrophil-mediated microbial killing	(184)
MPO	rs2333227	222	122/100	Portuguese	Not reported<br><br>Not reported	Genotypes<br><br>Genotypes	GG: 0.55 (0.32-0.94)<br>GA: 2.22 (1.26-3.89)	0.028<br><br>0.008		(185)
ORMDL 3	rs8076131	3022	1536/1486	Chinese Han	Not reported<br><br>Not reported	Alleles<br><br>Genotypes	1.15 (1.02-1.30)<br>1.43 (1.09-1.89)	0.02<br><br>0.01	Regulation of sphingolipid metabolism and inflammation	(187)
LTA	rs2239704	1976	1100/876	US	None	Alleles	1.31 (1.15-1.50)	0.013	Inflammatory signalling and	(195)

									immune cell activation	
LTA	rs1041981	451	320/131	Japanese	Not reported<br><br>Not reported	Genotypes<br><br>Genotypes	CA: 0.64 (0.40-1.02)<br>AA: 0.45 (0.21-0.95)	0.015<br><br>0.015		(196)
RAGE	rs2070600	1203	715/488	Chinese	Associated<br><br>Associated	Genotypes<br><br>Alleles	1.98 (1.42-2.75)<br>1.40 (1.19-1.65)	<0.001<br><br><0.001	Promoting cancer cell migration, differentiation and invasion	(189)
P2RX7	rs17525809	2126	1619/507	Taiwanese	Associated	Genotypes	10.2 (1.39-87.8)	0.03	ATP-mediated inflammatory signalling	(190)
HMGB1	rs1412125<br><br><br><br>rs2249825	417	305/112	Taiwanese	Not reported<br><br>Not reported<br><br>Not reported<br><br>Not reported	Genotypes<br><br>Genotypes<br><br>Genotypes<br><br>Genotypes	TC: 1.85 (1.12-3.04)<br>TC/CC: 1.99 (1.24-3.23)<br>CG: 2.04 (1.22-3.32)<br>CG/GG: 2.02 (1.22-3.32)	0.016<br><br>0.005<br><br>0.006<br><br>0.006	Inflammatory signalling and immune activation	(191)
ITPKC	rs28493229<br><br><br><br>rs28493229/rs890934/	1265	800/465	Taiwanese	None<br><br>None<br><br>None	Genotypes<br><br>Alleles<br><br>Haplotypes	1.18 (1.20-2.73)<br>1.70 (1.14-2.54)<br>CGTA: 0.59 (0.40-0.89)	0.005<br><br>0.008<br><br>0.01	Regulation of calcium signalling and immune cell activation	(192)

	rs2303723/rs10420685									
NFKB1	rs28362491	598	365/233	Chinese	Not reported	Genotypes	2.56 (1.46-4.49)	0.001	Transcriptional regulation of inflammation and immunity	(193)
						Alleles	1.49 (1.17-1.91)	0.001		
MIF	rs5844572	397	147/250	Chinese	Not reported	Genotypes	5/7: 3.51 (1.17-10.53)	0.022	Regulation of inflammatory and immune responses	(194)
					Not reported	Genotypes	6/7: 3.02 (1.17-7.79)	0.019		
					Not reported	Genotypes	7/7: 5.63 (125.26-.29)	0.018		
					Not reported	Alleles	2.22 (1.40-3.51)	0.001		
FOXP3	rs2294021	700	350/350	Chinese	Associated	Genotypes	TC: 0.71 (0.51-0.99)	0.044	Regulatory T-cell development and immune tolerance	(197)
					Associated	Genotypes	TC+CC: 0.69 (0.50-0.95)	0.022		
					Associated	Alleles	0.78 (0.63-0.97)	0.047		
	rs3761549				Associated	Genotypes	GA: 0.70 (0.51-0.96)	0.026		
					Associated	Genotypes	GA+AA: 0.69 (0.51-0.94)	0.018		
					Associated	Alleles	0.75 (0.58-0.96)	0.023		
TGFB1	rs1800469	312	162/150	Indian	Not reported	Genotypes	0.95 (0.91-0.99)	0.04	Immune regulation	(198)

ZNRD1-AS1	rs3757328	3022	1536/1486	Chinese	Not reported	Genotypes	0.83 (0.70-0.99)	0.033	Regulation of host immune responses and virus-related cancerogenesis	(188)
	rs6940552				Not reported	Genotypes	GA: 0.82 (0.70-0.97)	0.021		
					Not reported	Genotypes	AA: 0.67 (0.48-0.95)	0.026		
	rs9261204				Not reported	Genotypes	GA: 0.84 (0.71-0.99)	0.032		
					Not reported	Genotypes	GG: 0.60 (0.42-0.85)	0.005		

Table S8. Summary of studies reporting no significant association between immune response gene polymorphisms and cervical cancer risk

Gene	Polymorphism	Sample size	Unaffected/ affected	Population	Association with HPV	Distribution	OR (95% CI)	*p*-value	Function	Ref
TLR3	rs3775290	400	200/200	North Indian	Not reported	Genotypes	1.47 (0.58-3.70)	0.416	Viral dsRNA recognition and activation of antiviral signalling	(199)
TLR9	rs352140	400	200/200	North Indian	Not reported	Genotypes	0.87 (0.46-1.65)	0.662	Recognition of unmethylated CpG viral DNA	(199)
IL-10	rs1800896	161	80/81	Croatian	Not reported	Genotypes	Not reported	0.467	Involvement in anti-inflammatory responses	(200)
IL-10	rs1800896	420	122/298	Argentine	None	Genotypes	0.56 (0.17-1.82)	0.338		(201)
IL-1B	rs16944	161	80/81	Croatian	Not reported	Genotypes	Not reported	0.161	Involvement in acute and chronic inflammation	(200)
IL-18	rs360719<br>rs1946518<br>rs360722<br>rs187238	1192	722/470	Taiwanese	None<br>None<br>None<br>None	Genotypes<br>Genotypes<br>Genotypes<br>Genotypes	0.82 (0.63-1.08)<br>1.07 (0.82-1.41)<br>0.83 (0.63-1.08)<br>0.80 (0.61-1.05)	0.34<br>0.42<br>0.38<br>0.15	IFN-γ production and enhancement of Th1 immune response	(202)

	rs549908				None	Genotypes	1.10 (0.86-1.39)	0.74		
TNF-α	rs361525<br><br>rs1800629	247	134/113	North Macedonian	None<br><br>None	Genotypes<br><br>Genotypes	Not reported<br><br>Not reported	0.75<br><br>0.36	Involvement in inflammation, immune cell activation and apoptosis	(203)
TNF-α	rs361525<br><br>rs1800629	420	122/298	Argentine	None<br><br>None	Genotypes<br><br>Genotypes	0.42 (0.16-1.07)<br>0.89 (0.47-1.70)	0.069<br><br>0.739		(201)
MICA	c.638T>C<br><br>c.752G>A	194	94/100	Northeastern Thai	None<br><br>None	Alleles<br><br>Alleles	0.51<br><br>0.54	0.55<br><br>0.59	Activation of immune cells via NKG2D binding	(204)
TGFBR2	rs3087465	134	93/41	Brazilian	Not reported	Genotypes	GG: 3.37 (0.90-15.14)	0.086	Regulation of TGF-β signalling	(205)
FOXP3	rs3761548	134	93/41	Brazilian	Not reported	Genotypes	AA: 1.63 (0.62-4.37)	0.319	Regulation of regulatory T-cell development	(205)
CCR5	rs333	349	185/164	Brazilian	None	Genotypes	Not reported	0.167	Chemokine-mediated cell migration	(206)
NKG2C	CNV	867	295/572	Spanish	None	Genotypes	Not reported	HSIL vs. controls: 0.624	Natural killer cell activation	(207)
NFKB1	rs28362491	334	171/163	Brazilian	None	Genotypes	del/del: 0.96 (0.16-5.60)	0.96	Production of the p105/p50 protein	(208)

NFKBIA	rs696	334	171/163	Brazilian	None	Genotypes	AA: 1.42 (0.28-7.20)	0.67	Expression of the inhibitory protein IkBα	(208)
